# Supplementary material for: High-resolution simulations reveal positive global warming feedback from Pacific low clouds
Source: Sci Adv. 2026 Jul 24;12(30):eaec8488. doi: 10.1126/sciadv.aec8488 (PMC13398498; doi:10.1126/sciadv.aec8488)
Supplement: Supplementary file 1 — Figs. S1 to S15 References [file sciadv.aec8488_sm.pdf]

Science Advances
AAAS

Supplementary Materials for

High-resolution simulations reveal positive global warming feedback from Pacific low clouds

Sheide Chammas *et al.*

Corresponding author: Sheide Chammas, sheide@google.com; Tapio Schneider, tapio@google.com

Sci. Adv. **12**, eaec8488 (2026)
DOI: 10.1126/sciadv.aec8488

This PDF file includes:

Figs. S1 to S15
References

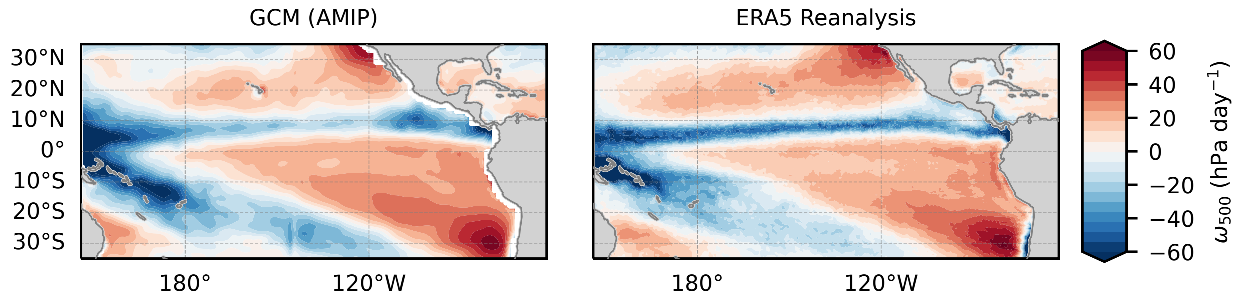

Figure S1: **Geographic distribution of the 500-hPa pressure velocity in the driving GCM and reanalysis** (hPa day^{-1})**, averaged over 2009–2014.** Positive values denote large-scale subsidence, characterizing marine low-cloud regimes, while negative values indicate ascent. The color scale magnitude is capped at 60 hPa day^{-1}. (Left) NOAA-GFDL CM4 model (AMIP simulation). (Right) ERA5 Reanalysis (*52*).

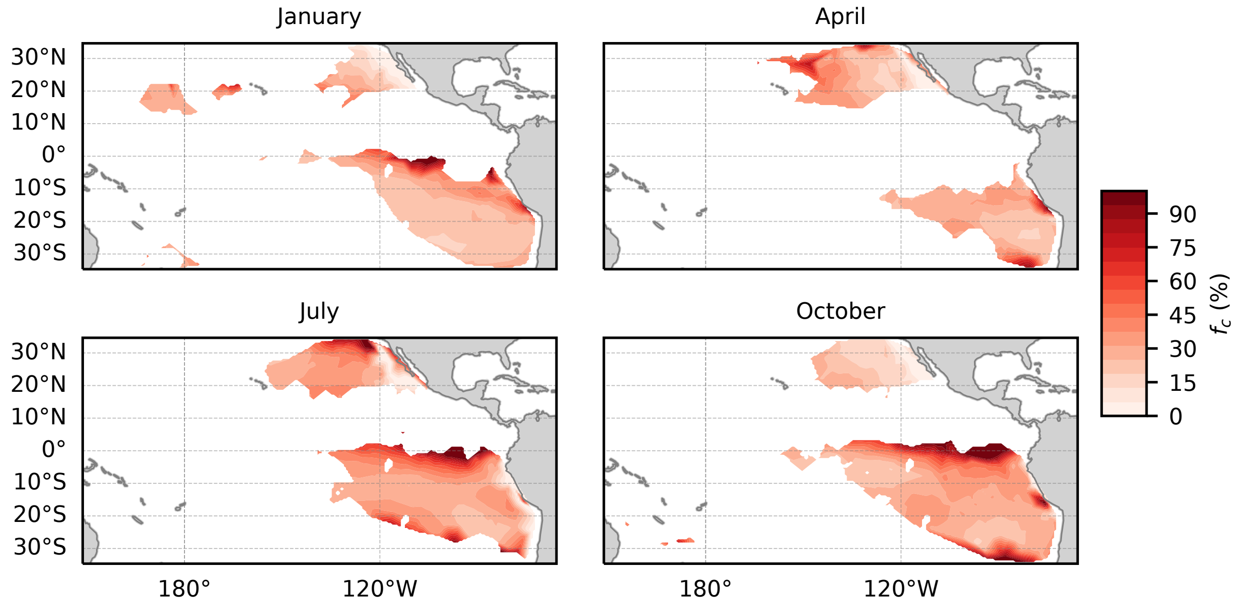

Figure S2: **Geographic distribution of seasonal cloud cover (**f_c**) simulated by LES in the baseline configuration.**

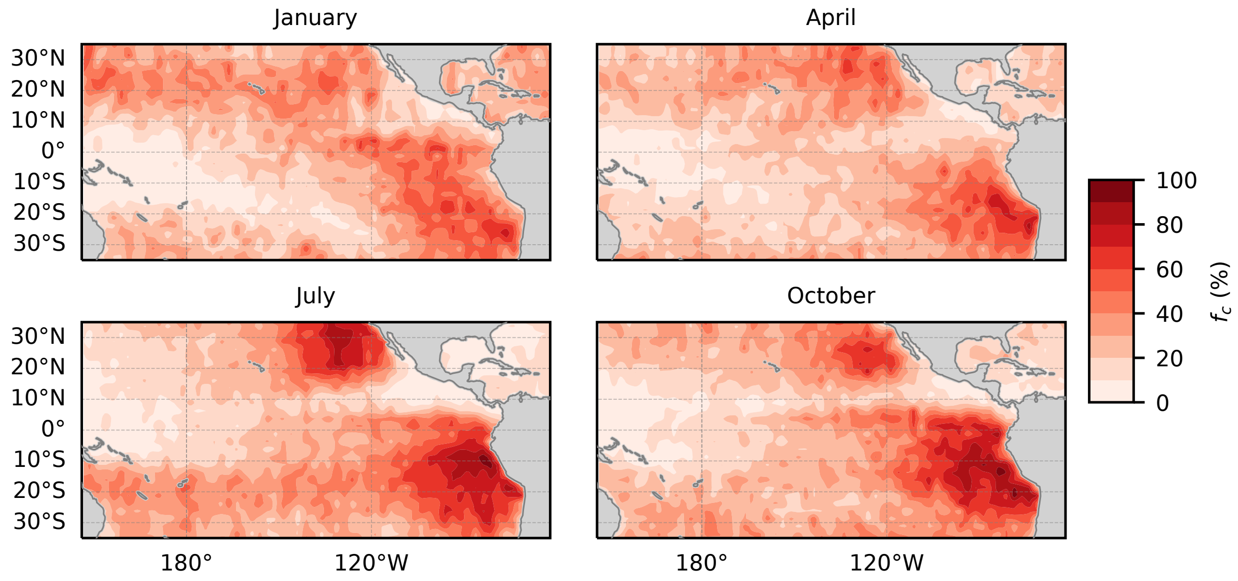

Figure S3: **Geographic distribution of seasonal low cloud cover (f_c) from merged observational data from two complementary satellite instruments (CloudSat and CALIPSO (*31*)) averaged over 2009-2014.**

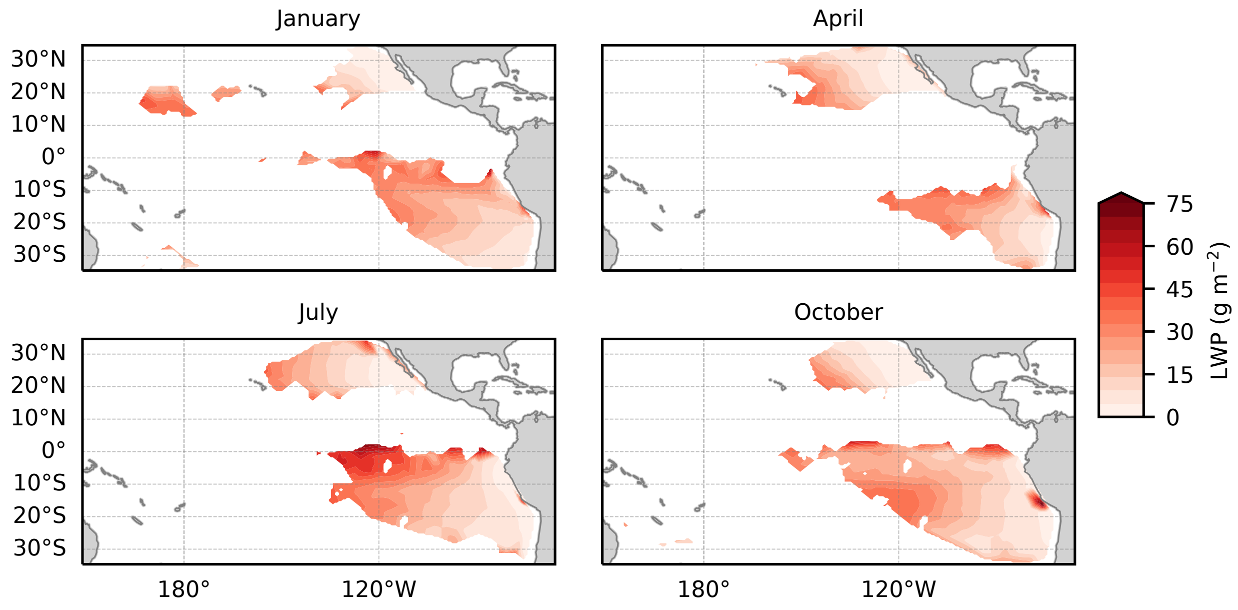

Figure S4: **Geographic distribution of the seasonal liquid water path (LWP, $\mathrm{g\,m^{-2}}$) simulated by LES in the baseline configuration.**

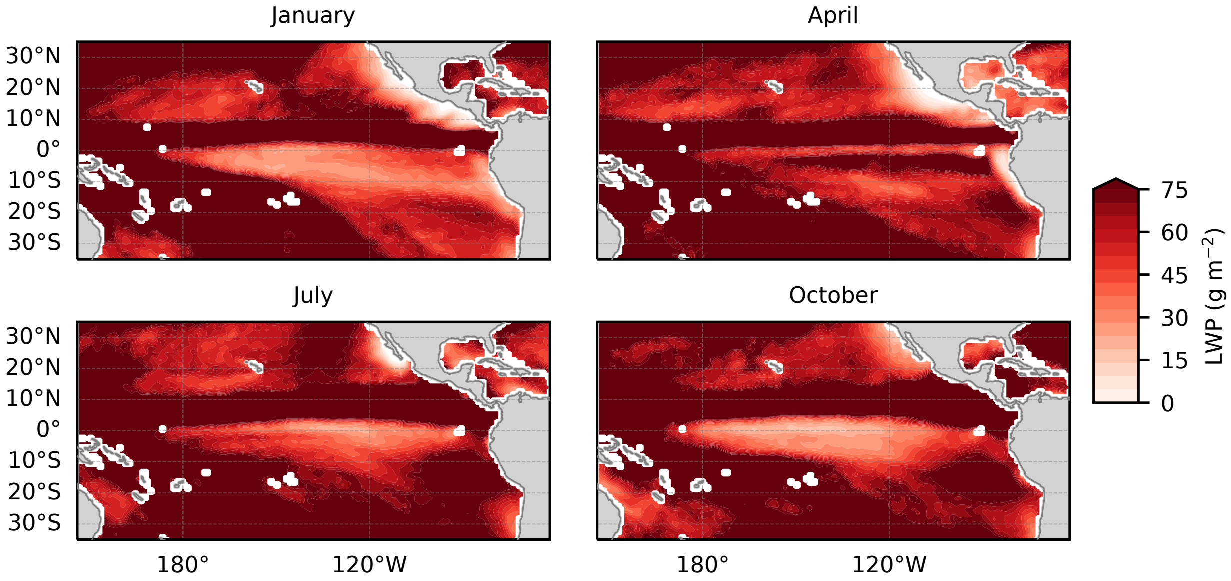

Figure S5: **Geographic distribution of the seasonal liquid water path (LWP,** $\mathrm{g\,m^{-2}}$**) from observational data from the MAC-LWP dataset (*55*) averaged over 2009–2014.** To emphasize spatial gradients and highlight variability within low-cloud regimes, the upper limit is capped at $75\,\mathrm{g\,m^{-2}}$, preventing these regional nuances from being obscured by the broad dynamic range of LWP across the broader Pacific.

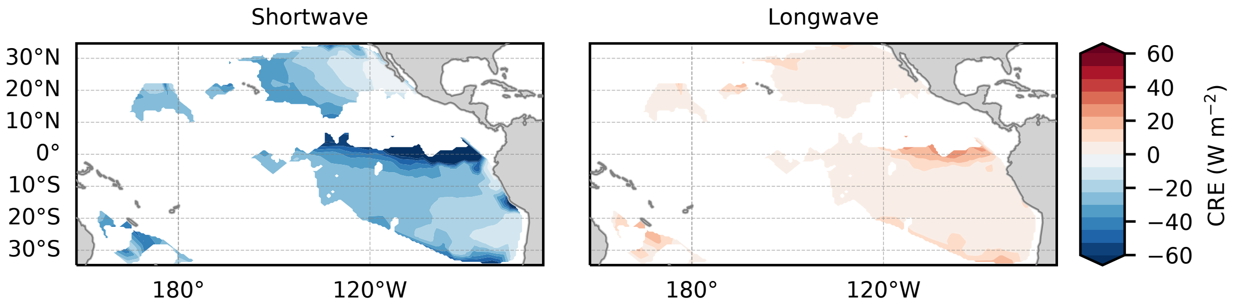

Figure S6: **Geographic distribution of the mean cloud radiative effect (CRE) in** W m^{-2} **simulated by LES in the baseline configuration.** (Left) Shortwave CRE. (Right) Longwave CRE. Negative SW values indicate a net cooling effect from the reflection of solar radiation by clouds, whereas positive LW values indicate a net warming effect from the trapping of outgoing thermal radiation by clouds. The values are averaged across all months.

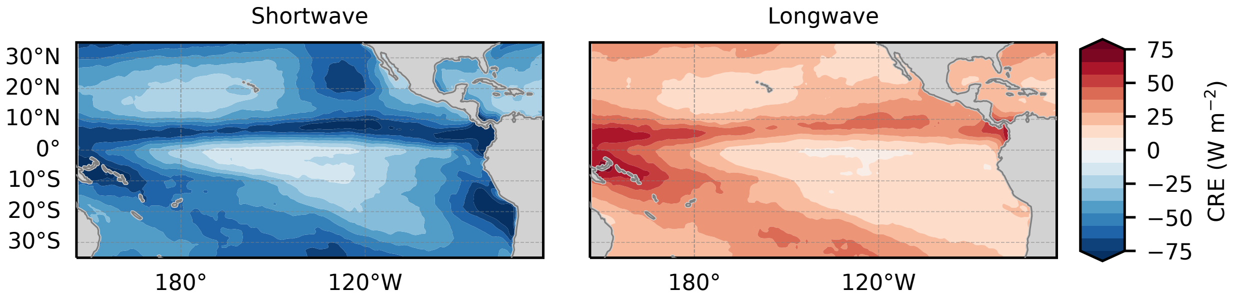

Figure S7: **Geographic distribution of the mean cloud radiative effect (CRE) in** W m^{-2} **from the CERES EBAF dataset (*56*) averaged over 2009–2014.** (Left) Shortwave CRE. (Right) Longwave CRE.

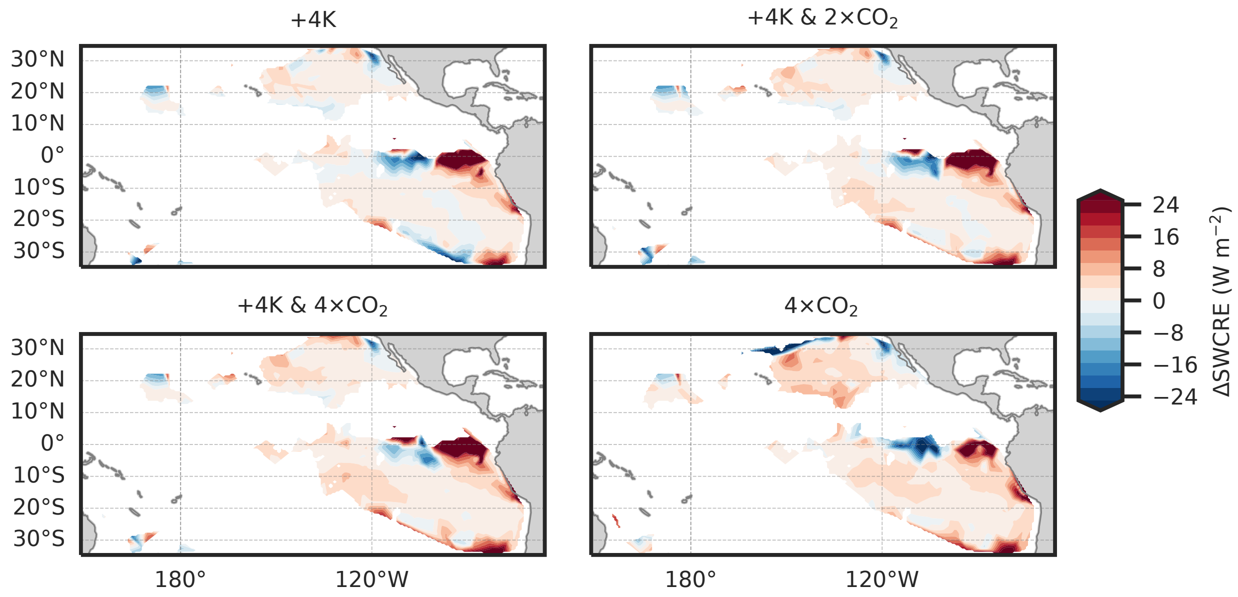

Figure S8: **Geographic distribution of changes in the shortwave cloud radiative effects (SWCRE) relative to the baseline.** Results are shown for the +4 K, +4 K+2×CO_2, +4 K+4×CO_2, and 4×CO_2 climate perturbation scenarios relative to the baseline, averaged across all months.

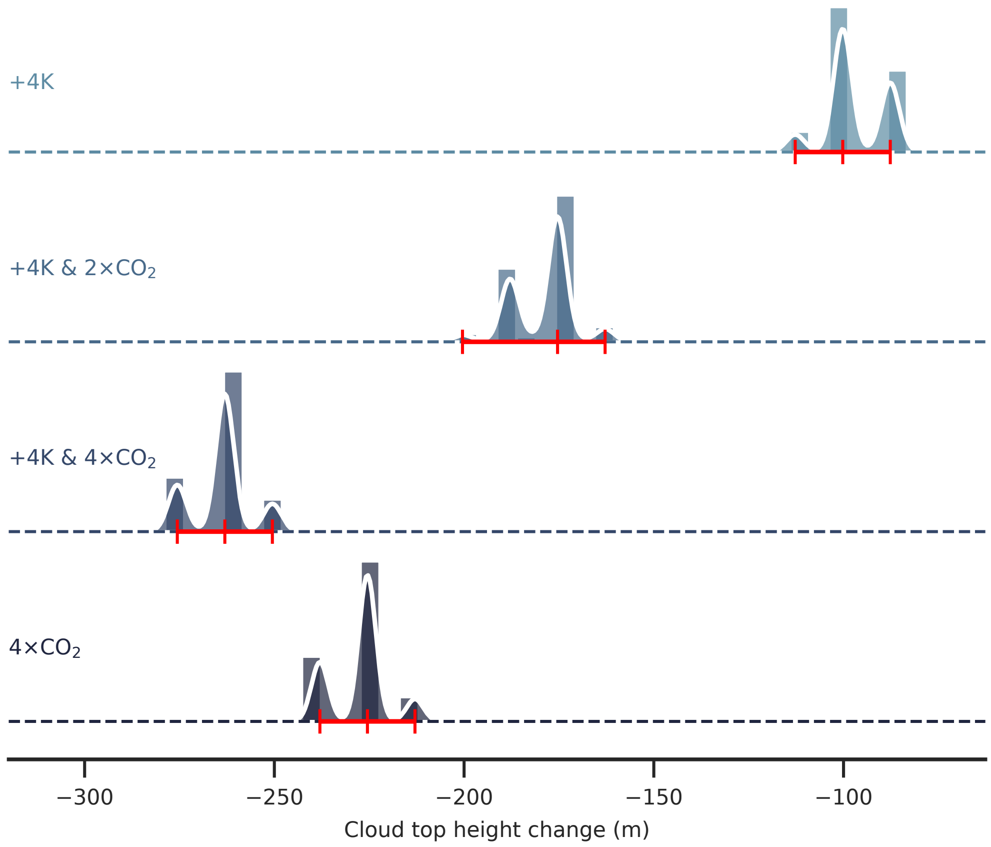

Figure S9: **Bootstrap distributions of the median changes in cloud top height relative to the baseline.** The cloud-top height distribution changes are widespread across the LES locations (Fig. S10).

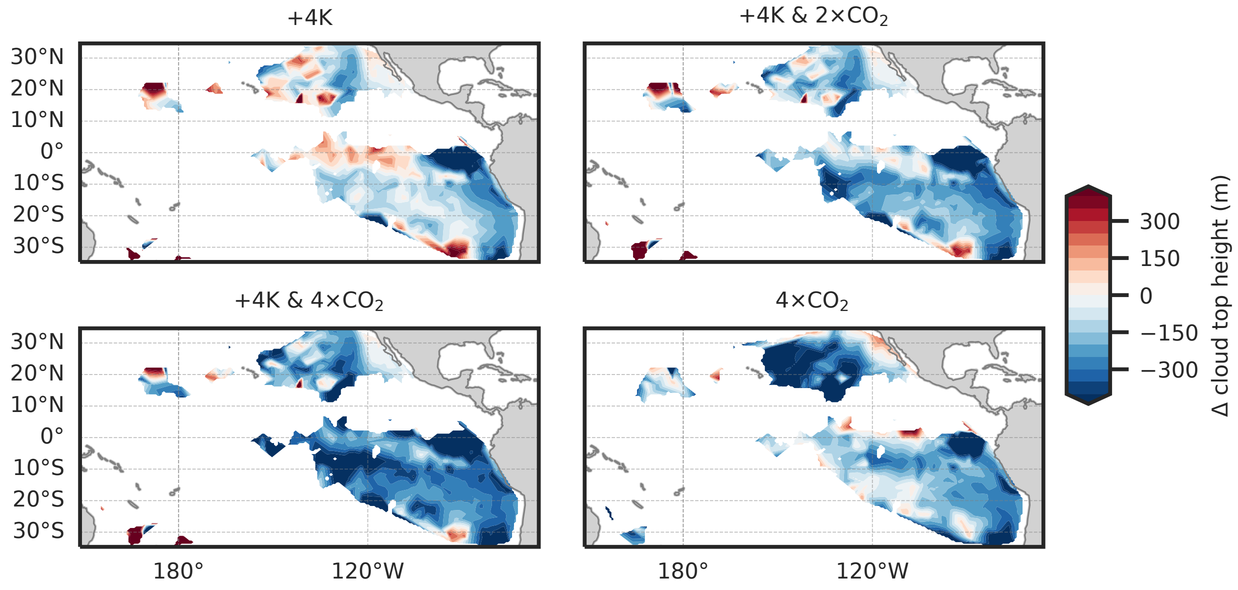

Figure S10: **Geographic distribution of changes in the cloud top height relative to the baseline.** Results are shown for the +4 K, +4 K+2× CO_2, +4 K+4× CO_2, and 4× CO_2 climate perturbation scenarios relative to the baseline, averaged across all months.

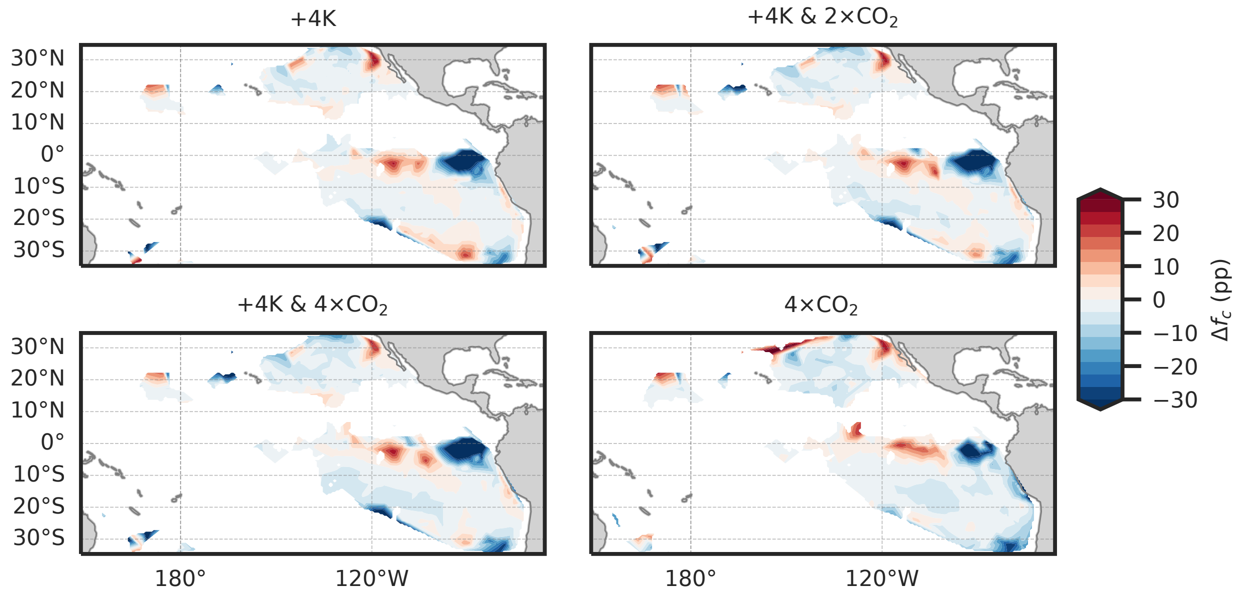

Figure S11: **Geographic distribution of changes in the mean cloud cover (f_c) relative to the baseline.** Results are shown for the +4 K, +4 K+2× CO_2, +4 K+4× CO_2, and 4× CO_2 climate perturbation scenarios.

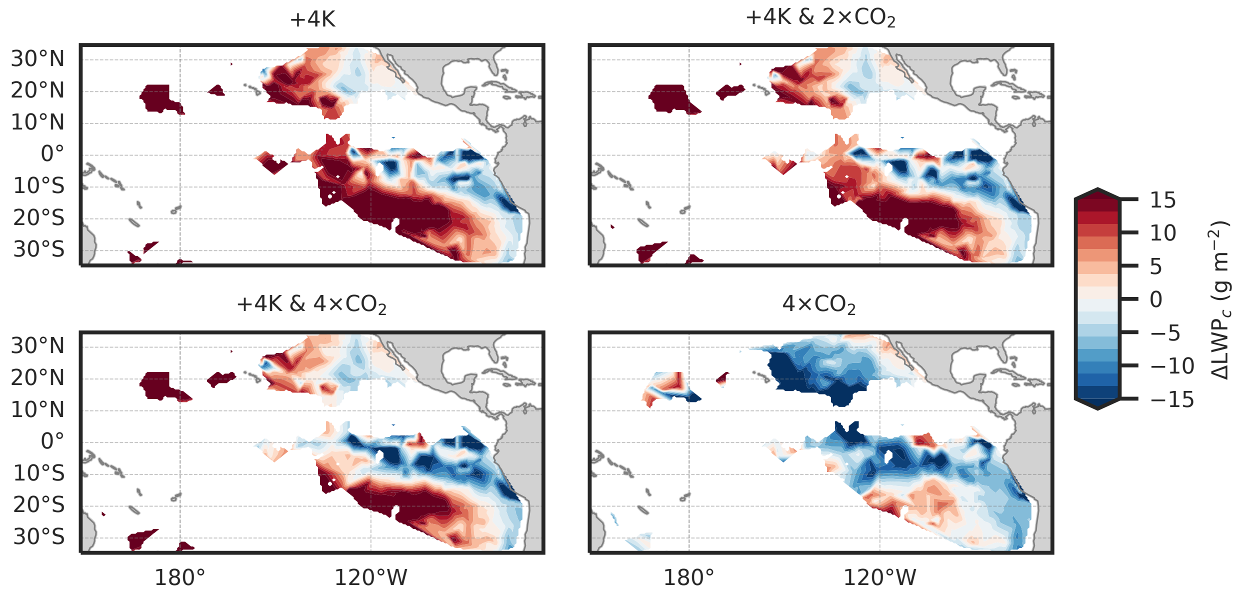

Figure S12: **Geographic distribution of changes in the in-cloud liquid water path** (LWP_c) **relative to the baseline.** Results are shown for the +4 K, +4 K+2×CO_2, +4 K+4×CO_2, and 4×CO_2 climate perturbation scenarios.

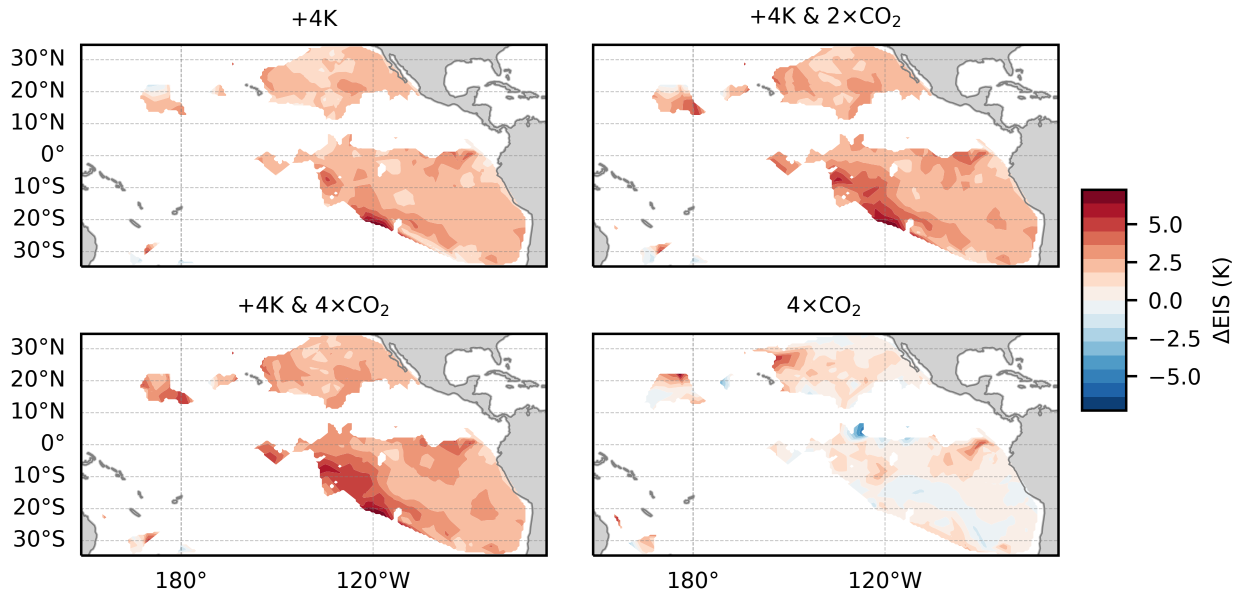

Figure S13: **Geographic distribution of changes in the estimated inversion strength (EIS) relative to the baseline.** Results are shown for the +4 K, +4 K+2×CO_2, +4 K+4×CO_2, and 4×CO_2 climate perturbation scenarios. The EIS changes are primarily driven by the host GCM dynamics.

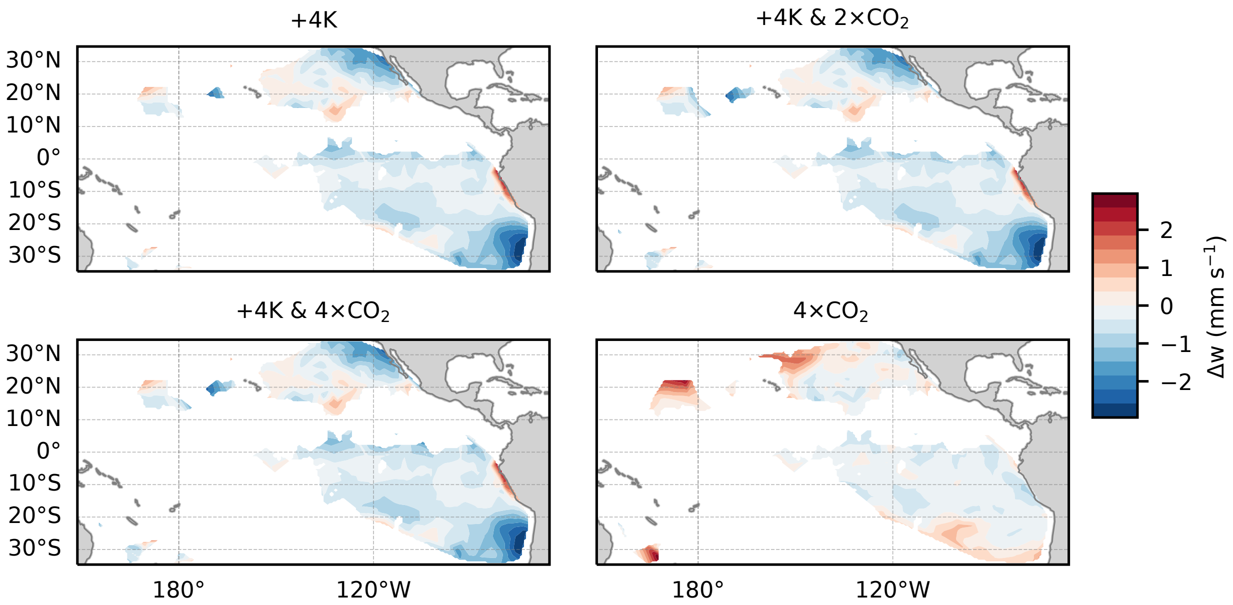

Figure S14: **Geographic distribution of changes in the mid-tropospheric large-scale subsidence at 4 km altitude (*w*) relative to the baseline, with negative values indicating subsidence weakening.** Results are shown for the +4 K, +4 K+2×CO_2, +4 K+4×CO_2, and 4×CO_2 climate perturbation scenarios. The subsidence changes are those of the host GCM.

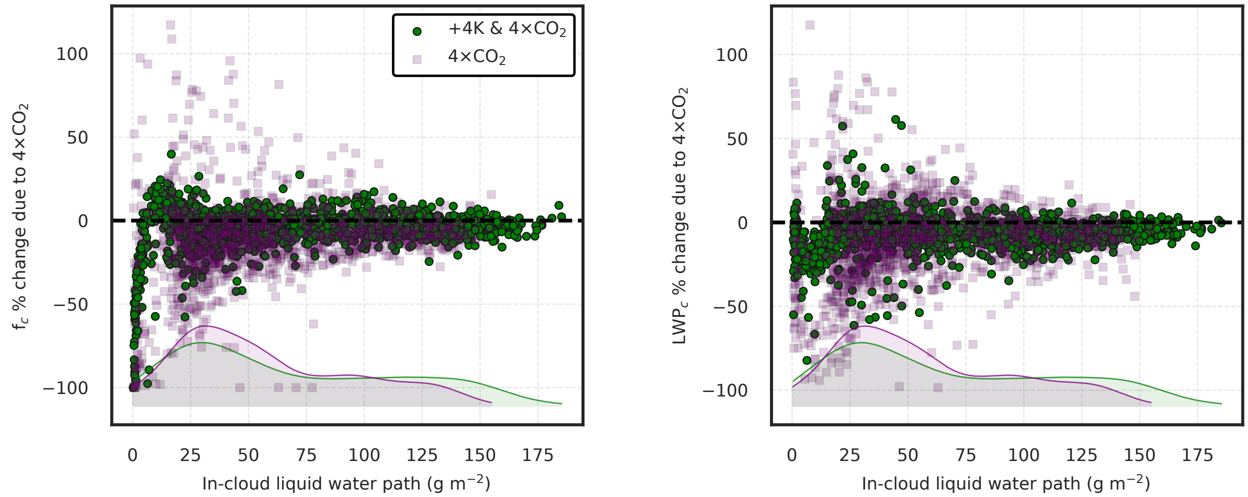

Figure S15: **Fractional changes in cloud properties under 4×CO_2 perturbations relative to the present-day climate and relative to +4 K.** (Left) Cloud cover change versus in-cloud liquid water path (LWP_c) of the base state. (Right) In-cloud liquid water path change versus LWP_c of the base state. Symbols distinguish between a 4×CO_2 increase relative to the present-day climate (purple squares) and relative to the +4 K scenario (green circles). The x-axis represents LWP_c both in the present-day climate (for purple circles) and in the +4 K scenario (for green circles). Overlaid on the bottom of the scatter plots are kernel density estimates (KDE) of the LWP_c distributions for the baseline climate (purple) and the +4 K scenario (green).

REFERENCES

1. F. A.-M. Bender, R. J. Charlson, A. M. Ekman, L. V. Leahy, Quantification of monthly mean regional-scale albedo of marine stratiform clouds in satellite observations and GCMs. *J. Appl. Meteorol. Climatol.* **50**, 2139–2148 (2011).

2. S. Bony, J. L. Dufresne, Marine boundary layer clouds at the heart of tropical cloud feedback uncertainties in climate models. *Geophys. Res. Lett.* **32**, L20806 (2005).

3. J. Vial, J.-L. Dufresne, S. Bony, On the interpretation of inter-model spread in CMIP5 climate sensitivity estimates. *Climate Dynam.* **41**, 3339–3362 (2013).

4. T. Schneider, J. Teixeira, C. S. Bretherton, F. Brient, K. G. Pressel, C. Schär, A. P. Siebesma, Climate goals and computing the future of clouds. *Nat. Clim. Chang.* **7**, 3–5 (2017).

5. G. L. Stephens, Cloud feedbacks in the climate system: A critical review. *J. Climate* **18**, 237–273 (2005).

6. S. C. Sherwood, M. J. Webb, J. D. Annan, K. C. Armour, P. M. Forster, J. C. Hargreaves, G. Hegerl, S. A. Klein, K. D. Marvel, E. J. Rohling, M. Watanabe, T. Andrews, P. Braconnot, C. S. Bretherton, G. L. Foster, Z. Hausfather, A. S. von der Heydt, R. Knutti, T. Mauritsen, J. R. Norris, C. Proistosescu, M. Rugenstein, G. A. Schmidt, K. B. Tokarska, M. D. Zelinka, An assessment of Earth's climate sensitivity using multiple lines of evidence. *Rev. Geophys.* **58**, e2019RG000678 (2020).

7. P. Forster, T. Storelvmo, K. Armour, W. Collins, J.-L. Dufresne, D. Frame, D.J. Lunt, T. Mauritsen, M.D. Palmer, M. Watanabe, M. Wild, H. Zhang, The Earth's energy budget, climate feedbacks, and climate sensitivity, in *Climate Change 2021: The Physical Science Basis. Contribution of Working Group I to the Sixth Assessment Report of the Intergovernmental Panel on Climate Change*, V. Masson-Delmotte, P. Zhai, A. Pirani, S.L. Connors, C. Péan, S. Berger, N. Caud, Y. Chen, L. Goldfarb, M.I. Gomis, M. Huang, K. Leitzell, E. Lonnoy, J.B.R. Matthews, T.K. Maycock, T. Waterfield, O. Yelekçi, R. Yu, B. Zhou, Eds. (Cambridge Univ. Press, 2021), pp. 923–1054.

8. M. D. Zelinka, T. A. Myers, D. T. McCoy, S. Po-Chedley, P. M. Caldwell, P. Ceppi, S. A. Klein, K. E. Taylor, Causes of higher climate sensitivity in CMIP6 models. *Geophys. Res. Lett.* **47**, e2019GL085782 (2020).

9. T. A. Myers, R. C. Scott, M. D. Zelinka, S. A. Klein, J. R. Norris, P. M. Caldwell, Observational constraints on low cloud feedback reduce uncertainty of climate sensitivity. *Nat. Clim. Chang.* **11**, 501–507 (2021).

10. G. V. Cesana, A. D. Del Genio, Observational constraint on cloud feedbacks suggests moderate climate sensitivity. *Nat. Clim. Chang.* **11**, 213–218 (2021).

11. M. D. Zelinka, S. A. Klein, Y. Qin, T. A. Myers, Evaluating climate models' cloud feedbacks against expert judgment. *J. Geophys. Res. Atmos.* **127**, e2021JD035198 (2022).

12. F. Brient, T. Schneider, Z. Tan, S. Bony, X. Qu, A. Hall, Shallowness of tropical low clouds as a predictor of climate models' response to warming. *Climate Dynam.* **47**, 433–449 (2016).

13. P. M. Caldwell, M. D. Zelinka, K. E. Taylor, K. Marvel, Quantifying the sources of intermodel spread in equilibrium climate sensitivity. *J. Climate* **29**, 513–524 (2016).

14. L.-W. Chao, M. D. Zelinka, A. E. Dessler, Evaluating cloud feedback components in observations and their representation in climate models. *J. Geophys. Res. Atmos.* **129**, e2023JD039427 (2024).

15. P. Ceppi, T. A. Myers, P. Nowack, C. J. Wall, M. D. Zelinka, Implications of a pervasive climate model bias for low-cloud feedback. *Geophys. Res. Lett.* **51**, e2024GL110525 (2024).

16. M. Zhang, C. S. Bretherton, P. N. Blossey, P. H. Austin, J. T. Bacmeister, S. Bony, F. Brient, S. K. Cheedela, A. Cheng, A. D. Del Genio, S. R. de Roode, S. Endo, C. N. Franklin, J. C. Golaz, C. Hannay, T. Heus, F. A. Isotta, J. L. Dufresne, I. S. Kang, H. Kawai, M. Köhler, V. E. Larson, Y. Liu, A. P. Lock, U. Lohmann, M. F. Khairoutdinov, A. M. Molod, R. A. J. Neggers, P. Rasch, I. Sandu, R. Senkbeil, A. P. Siebesma, C. Siegenthaler-le Drian, B. Stevens, M. J. Suarez, K. M. Xu, K. von Salzen, M. J. Webb, A. Wolf, M. Zhao, CGILS: Results from the first phase of an

international project to understand the physical mechanisms of low cloud feedbacks in general circulation models. *J. Adv. Model. Earth Syst.* **5**, 826–842 (2013).

17. C. S. Bretherton, P. N. Blossey, C. R. Jones, Mechanisms of marine low cloud sensitivity to idealized climate perturbations: A single-LES exploration extending the CGILS cases. *J. Adv. Model. Earth Syst.* **5**, 316–337 (2013).

18. P. N. Blossey, C. S. Bretherton, A. Cheng, S. Endo, T. Heus, A. P. Lock, J. J. van der Dussen, CGILS Phase 2 LES intercomparison of response of subtropical marine low cloud regimes to CO_2 quadrupling and a CMIP3 composite forcing change. *J. Adv. Model. Earth Syst.* **8**, 1714–1726 (2016).

19. Z. Tan, T. Schneider, J. Teixeira, K. G. Pressel, Large-eddy simulation of subtropical cloud-topped boundary layers: 2. Cloud response to climate change. *J. Adv. Model. Earth Syst.* **9**, 19–38 (2017).

20. P. G. Hill, D. L. Finney, M. D. Zelinka, Cloud feedback uncertainty in the equatorial Pacific across CMIP6 models. *Geophys. Res. Lett.* **52**, e2025GL117183 (2025).

21. J. Gregory, M. Webb, Tropospheric adjustment induces a cloud component in CO_2 forcing. *J. Clim.* **21**, 58–71 (2008).

22. M. D. Zelinka, S. A. Klein, K. E. Taylor, T. Andrews, M. J. Webb, J. M. Gregory, P. M. Forster, Contributions of different cloud types to feedbacks and rapid adjustments in CMIP5. *J. Climate* **26**, 5007–5027 (2013).

23. C. S. Bretherton, P. N. Blossey, Low cloud reduction in a greenhouse-warmed climate: Results from Lagrangian LES of a subtropical marine cloudiness transition. *J. Adv. Model. Earth Syst.* **6**, 91–114 (2014).

24. C. S. Bretherton, Insights into low-latitude cloud feedbacks from high-resolution models. *Philos. Trans. A Math. Phys. Eng. Sci.* **373**, 20140415 (2015).

25. M. Wu, H. Su, J. D. Neelin, Multi-objective observational constraint of tropical Atlantic and Pacific low-cloud variability narrows uncertainty in cloud feedback. *Nat. Commun.* **16**, 218 (2025).

26. S. Chammas, Q. Wang, T. Schneider, M. Ihme, Y.-f. Chen, J. Anderson, Accelerating large-eddy simulations of clouds with tensor processing units. *J. Adv. Model. Earth Syst.* **15**, e2023MS003619 (2023).

27. Z. Shen, A. Sridhar, Z. Tan, A. Jaruga, T. Schneider, A library of large-eddy simulations forced by global climate models. *J. Adv. Model. Earth Syst.* **14**, e2021MS002631 (2022).

28. M. Zhao, J.-C. Golaz, I. M. Held, H. Guo, V. Balaji, R. Benson, J.-H. Chen, X. Chen, L. J. Donner, J. P. Dunne, K. Dunne, J. Durachta, S.-M. Fan, S. M. Freidenreich, S. T. Garner, P. Ginoux, L. M. Harris, L. W. Horowitz, J. P. Krasting, A. R. Langenhorst, Z. Liang, P. Lin, S.-J. Lin, S. L. Malyshev, E. Mason, P. C. D. Milly, Y. Ming, V. Naik, F. Paulot, D. Paynter, P. Phillipps, A. Radhakrishnan, V. Ramaswamy, T. Robinson, D. Schwarzkopf, C. J. Seman, E. Shevliakova, Z. Shen, H. Shin, L. G. Silvers, J. R. Wilson, M. Winton, A. T. Wittenberg, B. Wyman, B. Xiang, The GFDL global atmosphere and land model AM4.0/LM4.0: 1. Simulation characteristics with prescribed SSTs. *J. Adv. Model. Earth Syst.* **10**, 691–734 (2018).

29. M. Zhao, J.-C. Golaz, I. M. Held, H. Guo, V. Balaji, R. Benson, J.-H. Chen, X. Chen, L. J. Donner, J. P. Dunne, K. Dunne, J. Durachta, S.-M. Fan, S. M. Freidenreich, S. T. Garner, P. Ginoux, L. M. Harris, L. W. Horowitz, J. P. Krasting, A. R. Langenhorst, Z. Liang, P. Lin, S.-J. Lin, S. L. Malyshev, E. Mason, P. C. D. Milly, Y. Ming, V. Naik, F. Paulot, D. Paynter, P. Phillipps, A. Radhakrishnan, V. Ramaswamy, T. Robinson, D. Schwarzkopf, C. J. Seman, E. Shevliakova, Z. Shen, H. Shin, L. G. Silvers, J. R. Wilson, M. Winton, A. T. Wittenberg, B. Wyman, B. Xiang, The GFDL global atmosphere and land model AM4.0/LM4.0: 2. Model description, sensitivity studies, and tuning strategies. *J. Adv. Model. Earth Syst.* **10**, 735–769 (2018).

30. K. A. Schiro, H. Su, F. Ahmed, N. Dai, C. E. Singer, P. Gentine, G. S. Elsaesser, J. H. Jiang, Y.-S. Choi, J. David Neelin, Model spread in tropical low cloud feedback tied to overturning circulation response to warming. *Nat. Commun.* **13**, 7119 (2022).

31. W. Bertrand, J. E. Kay, J. Haynes, G. de Boer, A global gridded dataset for cloud vertical structure from combined CloudSat and CALIPSO observations. *Earth Syst. Sci. Data* **16**, 1301–1316 (2024).

32. R. C. Scott, T. A. Myers, J. R. Norris, M. D. Zelinka, S. A. Klein, M. Sun, D. R. Doelling, Observed sensitivity of low-cloud radiative effects to meteorological perturbations over the global oceans. *J. Climate* **33**, 7717–7734 (2020).

33. G. L. Foster, D. L. Royer, D. J. Lunt, Future climate forcing potentially without precedent in the last 420 million years. *Nat. Commun.* **8**, 14845 (2017).

34. E. J. Judd, J. E. Tierney, D. J. Lunt, I. P. Montañez, B. T. Huber, S. L. Wing, P. J. Valdes, A 485-million-year history of Earth's surface temperature. *Science* **385**, eadk3705 (2024).

35. R. T. Wetherald, S. Manabe, Cloud feedback processes in a general circulation model. *J. Atmos. Sci.* **45**, 1397–1416 (1988).

36. B. J. Soden, I. M. Held, R. Colman, K. M. Shell, J. T. Kiehl, C. A. Shields, Quantifying climate feedbacks using radiative kernels. *J. Clim.* **21**, 3504–3520 (2008).

37. L. Peng, P. N. Blossey, W. M. Hannah, C. S. Bretherton, C. R. Terai, A. M. Jenney, S. L. Ferretti, H. Parishani, M. S. Pritchard, Resolving low cloud feedbacks globally with E3SM High-Res MMF: Agreement with LES but stronger shortwave effects. *J. Adv. Model. Earth Syst.* **17**, e2025MS005003 (2025).

38. T. Schneider, C. M. Kaul, K. G. Pressel, Possible climate transitions from breakup of stratocumulus decks under greenhouse warming. *Nat. Geosci.* **12**, 163–167 (2019).

39. T. Schneider, C. M. Kaul, K. G. Pressel, Solar geoengineering may not prevent strong warming from direct effects of CO_2 on stratocumulus cloud cover. *Proc. Natl. Acad. Sci. U.S.A.* **117**, 30179–30185 (2020).

40. M. Winton, K. Takahashi, I. M. Held, Importance of ocean heat uptake efficacy to transient climate change. *J. Clim.* **23**, 2333–2344 (2010).

41. T. Andrews, J. M. Gregory, D. Paynter, L. G. Silvers, C. Zhou, T. Mauritsen, M. J. Webb, K. C. Armour, P. M. Forster, H. Titchner, Accounting for changing temperature patterns increases historical estimates of climate sensitivity. *Geophys. Res. Lett.* **45**, 8490–8499 (2018).

42. T. A. Myers, M. D. Zelinka, S. A. Klein, Observational constraints on the cloud feedback pattern effect. *J. Climate* **36**, 6533–6545 (2023).

43. Y. Kamae, M. Watanabe, T. Ogura, M. Yoshimori, H. Shiogama, Rapid adjustments of cloud and hydrological cycle to increasing CO_2: A review. *Curr. Clim. Change Rep.* **1**, 103–113 (2015).

44. E. Kessler, *On the Distribution and Continuity of Water Substance in Atmospheric Circulations* (American Meteorological Society, 1969), vol. **10**.

45. Climate Modeling Alliance, CloudMicrophysics.jl [Software] (GitHub, 2025); https://github.com/CliMA/CloudMicrophysics.jl.

46. S. Azimi, A. Jaruga, E. K. de Jong, S. Arabas, T. Schneider, Training warm-rain bulk microphysics schemes using super-droplet simulations. *J. Adv. Model. Earth Syst.* **16**, e2023MS004028 (2024).

47. J.-P. Chen, T.-W. Hsieh, Y.-C. Lin, C.-K. Yu, Accurate parameterization of precipitation particles' fall speeds for bulk cloud microphysics schemes. *Atmos. Res.* **273**, 106171 (2022).

48. R. Pincus, E. J. Mlawer, J. S. Delamere, Balancing accuracy, efficiency, and flexibility in radiation calculations for dynamical models. *J. Adv. Model. Earth Syst.* **11**, 3074–3089 (2019).

49. Y. Liu, J. Hallett, The '1/3' power law between effective radius and liquid-water content. *Q. J. R. Meteorol. Soc.* **123**, 1789–1795 (1997).

50. R. L. Atlas, C. S. Bretherton, P. N. Blossey, A. Gettelman, C. Bardeen, P. Lin, Y. Ming, How well do large-eddy simulations and global climate models represent observed boundary layer structures and low clouds over the summertime Southern Ocean? *J. Adv. Model. Earth Syst.* **12**, e2020MS002205 (2020).

51. D. R. Durran, J. B. Klemp, A compressible model for the simulation of moist mountain waves. *Mon. Weather Rev.* **111**, 2341–2361 (1983).

52. H. Hersbach, B. Bell, P. Berrisford, S. Hirahara, A. Horányi, J. Muñoz-Sabater, J. Nicolas, C. Peubey, R. Radu, D. Schepers, A. Simmons, C. Soci, S. Abdalla, X. Abellan, G. Balsamo, P. Bechtold, G. Biavati, J. Bidlot, M. Bonavita, G. de Chiara, P. Dahlgren, D. Dee, M. Diamantakis, R. Dragani, J. Flemming, R. Forbes, M. Fuentes, A. Geer, L. Haimberger, S. Healy, R. J. Hogan, E. Hólm, M. Janisková, S. Keeley, P. Laloyaux, P. Lopez, C. Lupu, G. Radnoti, P. de Rosnay, I. Rozum, F. Vamborg, S. Villaume, J.-N. Thépaut, The ERA5 global reanalysis. *Q. J. R. Meteorol. Soc.* **146**, 1999–2049 (2020).

53. B. J. Soden, A. J. Broccoli, R. S. Hemler, On the use of cloud forcing to estimate cloud feedback. *J. Clim.* **17**, 3661–3665 (2004).

54. R. Wood, C. S. Bretherton, On the relationship between stratiform low cloud cover and lower-tropospheric stability. *J. Clim.* **19**, 6425–6432 (2006).

55. G. S. Elsaesser, C. W. O'Dell, M. D. Lebsock, R. Bennartz, T. J. Greenwald, F. J. Wentz, The Multisensor Advanced Climatology of Liquid Water Path (MAC-LWP). *J. Clim.* **30**, 10193–10210 (2017).

56. N. G. Loeb, D. R. Doelling, H. Wang, W. Su, C. Nguyen, J. G. Corbett, L. Liang, C. Mitrescu, F. G. Rose, S. Kato, Clouds and the Earth's Radiant Energy System (CERES) Energy Balanced and Filled (EBAF) top-of-atmosphere (TOA) Edition-4.0 data product. *J. Climate* **31**, 895–918 (2018).
