# Supplementary material for: High-resolution simulations reveal positive global warming feedback from Pacific low clouds
Source: Sci Adv. 2026 Jul 24;12(30):eaec8488. doi: 10.1126/sciadv.aec8488 (PMC13398498; doi:10.1126/sciadv.aec8488)
Supplement: Supplementary file 2 [file sciadv.aec8488.v1.pdf]

ATMOSPHERIC SCIENCE

High-resolution simulations reveal positive global warming feedback from Pacific low clouds

Sheide Chammas[1]*, Qing Wang[1], Tapio Schneider[1,2]*, Rob Carver[1], Zhaoyi Shen[2], Jeffrey B. Parker[1], Cenk Gazen[1], Matthias Ihme[1,3], Yi-Fan Chen[1], John Anderson[1]

Copyright © 2026 The Authors, some rights reserved; exclusive licensee American Association for the Advancement of Science. No claim to original U.S. Government Works. Distributed under a Creative Commons Attribution License 4.0 (CC BY).

Uncertainty in marine low-level cloud feedbacks limits accurate climate projections. Using 7083 high-resolution simulations of tropical Pacific low clouds, we separated the impacts of sea surface warming from direct carbon dioxide (CO_2) effects. Surface warming alone drives a positive low-cloud feedback of 0.14 W m^{-2} K^{-1}. While this changes little with doubled CO_2, the total cloud radiative response strengthens markedly to 0.43 W m^{-2} K^{-1} under quadrupled CO_2, revealing a strong nonlinear interaction. Surface warming alone modifies the boundary layer through increased inversion strength and weakened subsidence, which buffers clouds by promoting higher liquid water content while cloud fraction decreases. Rapid adjustments to high CO_2 concentrations counteract this protective cloud thickening. Consequently, a pronounced reduction in cloud fraction is no longer offset by an increase in cloud brightness, markedly strengthening the total radiative response under quadrupled CO_2. Ultimately, our results suggest that climate sensitivity is more state-dependent than often assumed.

INTRODUCTION

Marine low-level clouds substantially cool Earth by reflecting up to about 40% of incoming sunlight (*1*), yet their response to climate change remains the largest source of uncertainty in climate projections (*2–9*). This uncertainty is a primary reason that global climate models (GCMs) disagree on the equilibrium climate sensitivity (ECS)—the eventual global warming reached after a sustained CO_2 doubling (*7*, *8*, *10*, *11*). GCMs cannot resolve the fine-scale turbulence governing these clouds (*12*, *13*); instead, they rely on simplified parameterizations that are a known source of model bias and intermodel spread (*3*, *4*, *14*, *15*).

To circumvent these limitations, we performed an unprecedented ensemble of more than 7000 high-resolution large-eddy simulations (LESs), which explicitly resolve cloud-scale dynamics. Whereas prior LES studies were limited to a few idealized cases (*16–19*), our simulations cover diverse meteorological conditions across the tropical Pacific, an important region for cloud feedback uncertainty (*20*). We systematically perturbed sea surface temperatures (SSTs) and CO_2 concentrations, both individually and in combination. This design allows us to isolate the cloud response to surface warming (the climate feedback) from the rapid cloud adjustments driven by the direct radiative effects of CO_2 (*21*, *22*). Separating these processes is essential because higher CO_2 concentrations can inhibit the cloud-top longwave (LW) cooling that sustains stratocumulus decks, thereby causing rapid cloud adjustments that alter cloud properties (*17*, *19*, *23*, *24*). Our analysis provides robust, process-based constraints on how these distinct mechanisms interact to shape the total cloud response to climate change. The extensive public dataset from these simulations provides a transformative new resource for evaluating models, developing and calibrating GCM parameterizations, and addressing model biases (*14*, *15*, *25*).

[1]Google Research, Mountain View, CA, USA. [2]California Institute of Technology, Pasadena, CA, USA. [3]Stanford University, Stanford, CA, USA.
*Corresponding author. Email: sheide@google.com (S.C.); tapio@google.com (T.S.)

RESULTS

Large-ensemble LES experiments

To investigate low-cloud responses, we generated an extensive ensemble of 7083 LESs, a scale made computationally feasible using an LES code that leverages Google's tensor processing unit clusters (*26*). Following previously established methodology (*27*), we forced the LES with large-scale conditions derived from a single GCM, the GFDL CM4 (*28*, *29*). The GFDL CM4 was chosen due to its well-documented skill in simulating present-day atmospheric dynamics and realistic baseline climatologies (e.g., mid-tropospheric vertical velocities compare well with reanalysis, with some notable differences in the northeastern subtropical Pacific, fig. S1); it is also one of the few models providing the necessary high-frequency output required for this forcing methodology. While using a single driving model is a limitation (*30*), prior research indicates that the differences in cloud feedbacks between GCMs are generally larger than those between LES driven by different GCMs (*27*). The forcing conditions were derived for 500 randomly selected locations across the tropical Pacific Ocean (35°S to 35°N) for four representative months (January, April, July, and October). These locations, shown in Fig. 1, were selected from regions with prevalent marine low-level clouds identified by satellite observations (*31*) and where time-averaged ERA5 reanalysis subsidence velocities ($\omega_{500\ \mathrm{hPa}} > 8$ hPa day^{-1}) indicate conditions less prone to deep convection. Driving the LES at these locations and times with output from an Atmospheric Model Intercomparison Project (AMIP) configuration of the GCM, representing today's climate, yields a diversity of simulated cloud fields, including stratocumulus, stratocumulus over cumulus, and cumulus (Fig. 1); while the simulated geographic distribution of cloud properties resembles observations (*32*) in some respects (e.g., dense stratocumulus decks off Peru), it differs in others, such as a westward increase of cloud cover across the North Pacific, and a general underestimation of LWP, at least against one reference dataset (figs. S2 to S7). Nonetheless, this ensemble provides a useful large sample for probing the cloud response to climate change.

Each location-season combination was used to drive LES for four climate change scenarios, in addition to the AMIP baseline: a 4 K SST increase relative to baseline (+4 K), a quadrupling of atmospheric

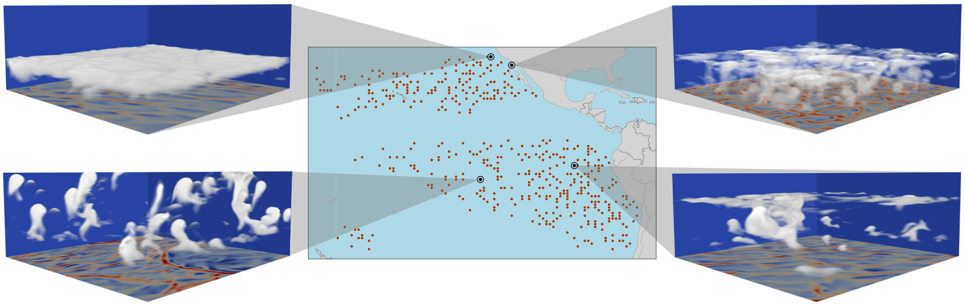

Fig. 1. Pacific locations sampled and representative cloud regimes in baseline LES. (**Middle**) The 500 Pacific locations sampled. (**Side panels**) Volumetric renderings of cloud water mass fraction in LES driven by GCM output for today's climate at 4 representative locations (black circles) during July, revealing distinct low-cloud regimes (clockwise from lower left: shallow cumulus, stratocumulus, coastal stratocumulus with fog, and stratocumulus over cumulus). The bottom plane renders surface buoyancy.

CO_2 alone (4×CO_2), and two scenarios combining 4 K warming with either doubled (+4 K + 2×CO_2) or quadrupled (+4 K + 4×CO_2) CO_2. While a quadrupling of CO_2 represents an extreme near-term forcing, it serves as a standard Climate Model Intercomparison Project (CMIP) benchmark to isolate and magnify the signal of direct radiative effects on cloud-top cooling. Furthermore, understanding this extreme state is relevant for modeling and understanding past hothouse climates, such as the Eocene, where CO_2 levels were up to four times higher than today (*33*, *34*). The experimental design includes large-scale thermal and circulation changes from the host GCM but excludes additional local feedbacks, relying instead on the feedback response of the LES itself. Our analysis focuses on low clouds, and we excluded simulations where clouds grew above 4-km altitude, yielding the 7083 LESs for analysis (see Materials and Methods). We quantified the top-of-atmosphere (TOA) radiative impact of cloud changes using the partial radiative perturbation (PRP) method (*35*, *36*), which isolates the TOA radiation change caused solely by substituting cloud fields from a perturbed state into the baseline environment; as a complement, we also computed the backward PRP obtained by substituting baseline cloud fields into the perturbed environment. The average of the forward and backward PRP radiative changes in the +4 K scenario, normalized by the surface warming, yields the low-cloud feedback. For scenarios with simultaneous SST and CO_2 changes, we also normalize the radiative impact by 4 K; we term this the total cloud radiative response, as it encompasses both the temperature-mediated feedback and rapid adjustments to CO_2 forcing (*21*, *22*). While separating the cloud response to temperature and direct CO_2 changes is helpful for mechanistic understanding, their combined effect, reflected in the total cloud radiative response, is what the climate system ultimately experiences.

Robust positive low-cloud feedback

Our LES ensemble reveals a strong, nonlinear cloud response when surface warming is combined with high concentrations of CO_2. In response to a 4 K surface warming alone (+4 K), changes in low clouds increase the net cloud radiative effect (CRE) at the top of the atmosphere—the difference between all-sky and clear-sky radiation—by 0.10 (0, 0.42) $W\,m^{-2}$ (median and 95% confidence interval; Fig. 2). This is driven primarily by reduced shortwave (SW) reflection, which increases the Earth system's SW absorption by 1.31 (0.97, 1.53) $W\,m^{-2}$, and is widespread across the domain (fig. S8). It is partially offset by a reduction in the LW greenhouse effect of the clouds [−0.94 (−1.01, −0.90) $W\,m^{-2}$]. A quadrupling of CO_2 without surface warming (4×CO_2) has an even stronger effect of 1.93 (1.73,2.04) $W\,m^{-2}$. This causes a large positive change in the CRE, primarily driven by rapid adjustments that decrease cloud fraction and liquid water path, thereby reducing SW reflection. When these forcings are combined (+4 K + 4×CO_2), however, the total radiative effect from cloud changes [0.77 (0.52, 0.95) $W\,m^{-2}$] is less than half the linear sum of the individual responses. This demonstrates a significant nonlinear interaction, with warming buffering the direct cloud-dissipating effect of increased CO_2 concentrations.

To systematically isolate the temperature-mediated feedbacks from cloud masking effects—such as the strong masking of SW reflection by increased atmospheric water vapor in the warmed climate—we calculate the cloud radiative response using the PRP method (Table 1). The total cloud radiative response demonstrates a pronounced nonlinear interaction when surface warming is combined with quadrupled CO_2 (Fig. 3). The warming-only response corresponds to a low-cloud feedback of 0.14 (0.02, 0.25) $W\,m^{-2}\,K^{-1}$ (median and 95% confidence interval). This feedback is robustly positive (95% confidence intervals exclude zero), indicating tropical low clouds amplify warming. The combined +4 K + 4×CO_2 scenario yields a total cloud radiative response of 0.43 (0.33, 0.53) $W\,m^{-2}\,K^{-1}$, while the intermediate +4 K + 2×CO_2 scenario is only slightly stronger than the warming-only case at 0.15 (0.05, 0.28) $W\,m^{-2}\,K^{-1}$. A decomposition into SW and LW components shows the nonlinear strengthening is SW-dominated (Fig. 3). The SW feedback to warming is strongly positive, while the LW feedback is weaker and negative, consistent with widespread reductions in cloud-top height under warming (figs. S9 and S10) (*17*–*19*, *37*). As detailed in Materials and Methods, our offline radiative calculations use GCM-derived thermodynamic profiles above the LES domain but exclude GCM high clouds. This idealization might overestimate the negative LW low-cloud feedback magnitude by omitting potential masking by overlying high clouds (e.g., cirrus), which may have weak SW but substantial LW effects. However, because the LW low-cloud feedback is already weak, masking by overlying high clouds is unlikely to affect the sign of the net feedback. The dominant-positive SW component results in a net-positive feedback, characteristic of low clouds whose primary radiative influence is due to reflection of solar radiation.

These LES-derived results offer important insights. Our net low-cloud feedback of 0.14 to 0.15 $W\,m^{-2}\,K^{-1}$ under 4 K warming over the Pacific is similar to recent observationally derived global estimates (*9*); however, our Pacific-only domain means one cannot directly scale up to a global feedback and that agreement with

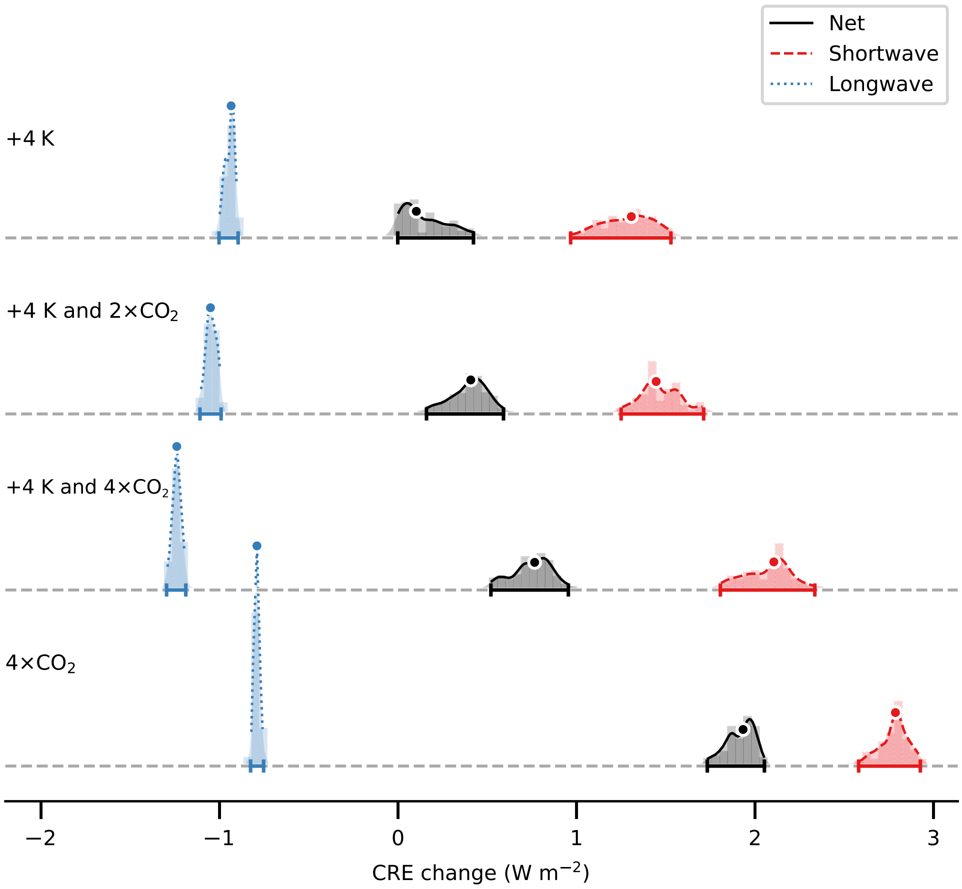

Fig. 2. Bootstrap distributions of the median changes in CRE relative to the baseline CRE. The perturbation scenarios are labeled on the left of each panel (+4 K, +4 K + 2×CO_2, +4 K + 4×CO_2, and 4×CO_2). Distributions show the uncertainty in the median change for net (black, solid line), SW (red, dashed line), and LW (blue, dotted line) CRE. Each distribution is obtained from 10,000 bootstrap resamples of the full LES ensemble. The 95% confidence interval for a given distribution is represented both by the truncation of its styled line and by the color-matched whiskered interval at the baseline of the panel. A white-outlined dot in the corresponding color marks the median of each bootstrap distribution.

Table 1. Summary of radiative responses. The median PRP results isolating the temperature-mediated feedbacks and rapid adjustments. For comparison, the corresponding mean PRP results are given in parentheses; the mean values are strongly influenced by influential outliers with extreme radiative responses and hence are larger in magnitude than the median values. (The individual median SW and LW components do not sum exactly to the median net response because of the nonlinearity of the median; however, the mean SW and LW components do sum to the net response, except for minor rounding differences).

Scenario	SW component (W m^{-2} K^{-1})	LW component (W m^{-2} K^{-1})	Net response (W m^{-2} K^{-1})
+4 K	0.18 (0.38)	−0.03 (−0.10)	0.14 (0.28)
+4 K + 2×CO_2	0.23 (0.59)	−0.02 (−0.14)	0.15 (0.45)
+4 K + 4×CO_2	0.49 (1.00)	−0.04 (−0.19)	0.43 (0.80)

the global estimates is therefore only suggestive. The SW portion (~0.2 W m^{-2} K^{-1}) is also comparable in sign and magnitude to estimates from recent global multiscale modeling framework (MMF) simulations (*37*), although we note our regional focus limits direct comparisons to global values. However, the much stronger total response with quadrupled CO_2 places our findings at the upper end of GCM-simulated values (*8*), highlighting the importance of accurately representing rapid CO_2-cloud interactions in models (*15*).

The nonlinearity is consistent with prior LES work showing that at roughly quadrupled CO_2, stratocumulus decks can destabilize and break up when direct radiative effects, combined with warming, diminish the cloud-top cooling that sustains stratocumulus decks sufficiently (*19*, *23*, *38*, *39*). The broader implication is that climate sensitivity may be more state-dependent than is often assumed, making the accurate representation of rapid cloud adjustments to CO_2 essential for robust climate projections.

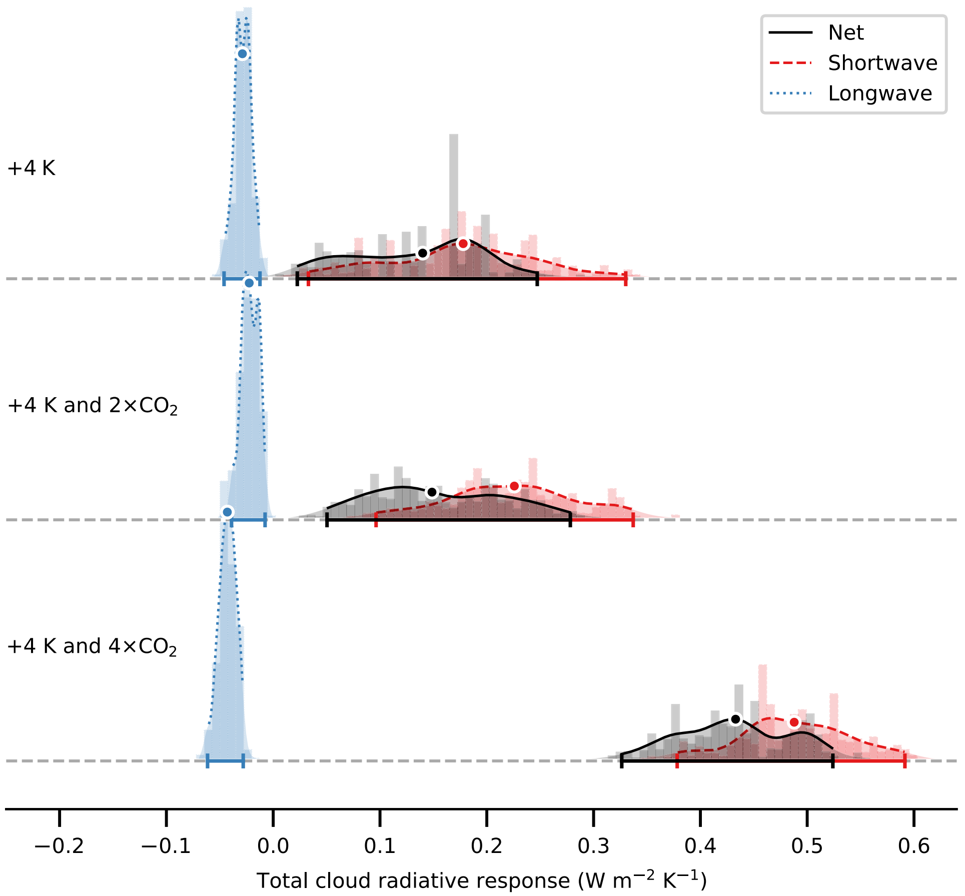

Fig. 3. Bootstrap distributions of the median net, SW, and LW total cloud radiative response for the three climate perturbation scenarios that include surface warming. Distributions show the uncertainty in the median net (black, solid line), SW (red, dashed line), and LW (blue, dotted line) total cloud radiative response. Each distribution is obtained from 10,000 bootstrap resamples of the full LES ensemble. The 95% confidence interval for a given distribution is represented both by the truncation of its styled line and by the color-matched whiskered interval at the baseline of the panel. A white-outlined dot in the corresponding color marks the median of each bootstrap distribution. A positive cloud feedback implies amplification of the warming through changes in cloud properties; a negative feedback indicates attenuation.

Cloud feedback mechanisms

The physical drivers of the radiative response involve competing changes in cloud fraction (f_c) and in-cloud liquid water path (LWP_c). A general decrease in low-cloud fraction, which reduces planetary albedo, is the primary driver of the positive SW response (Fig. 4, left panels) and extends over most of the simulated locations in all scenarios (fig. S11). This feature is common in both LES and global models under warming (*8*, *9*, *24*, *37*). In our +4 K warming simulations, however, this effect is partially offset by a robust increase in LWP_c (2.4 g m^{-2} in +4 K-only), which in isolation makes clouds optically thicker. This LWP_c increase is physically consistent with changes in large-scale environmental controls shown in our simulations (Fig. 5): a stronger estimated inversion strength (EIS) and weaker large-scale subsidence, both of which are known to promote higher LWP_c in the simulated conditions (*17*, *19*). This is also borne out in the spatial patterns of LWP_c changes: The cloud thickening in the +4 K simulations is enhanced in locations with increased EIS (cf. figs. S12 and S13), with a weaker spatial correlation with subsidence changes, which are particularly strong in coastal regions (fig. S14).

In contrast, the direct response to CO_2 drives a decrease in both f_c and LWP_c. In the 4×CO_2-only experiment, where changes to EIS and subsidence are minimal (Fig. 5), clouds exhibit a strong decrease in both fractional area coverage and liquid water content (median LWP_c change of −4.7 g m^{-2}). Our results show these rapid cloud adjustments to CO_2 drive particularly large fractional reductions in clouds that are already tenuous (baseline $LWP_c < 50$ g m^{-2}; fig. S15). In the combined +4 K + 4×CO_2 scenario, in addition to further reducing f_c, this rapid cloud adjustment to CO_2 negates the warming-induced LWP_c increase, resulting in a near-neutral change of −0.1 g m^{-2}. The resulting strong positive total response stems from this combination: a pronounced reduction in cloud area that is no longer opposed by an increase in cloud brightness (summarized conceptually in Fig. 6).

Last, our results reveal that the rapid cloud adjustment to CO_2 is strongly state dependent. The cloud-reducing impact of quadrupled CO_2 is significantly weaker when applied to the warmed +4 K climate than to the baseline climate. This occurs because the warming-induced environmental changes—the stronger EIS and weaker subsidence that stabilize the boundary layer (Fig. 5)—buffer the clouds.

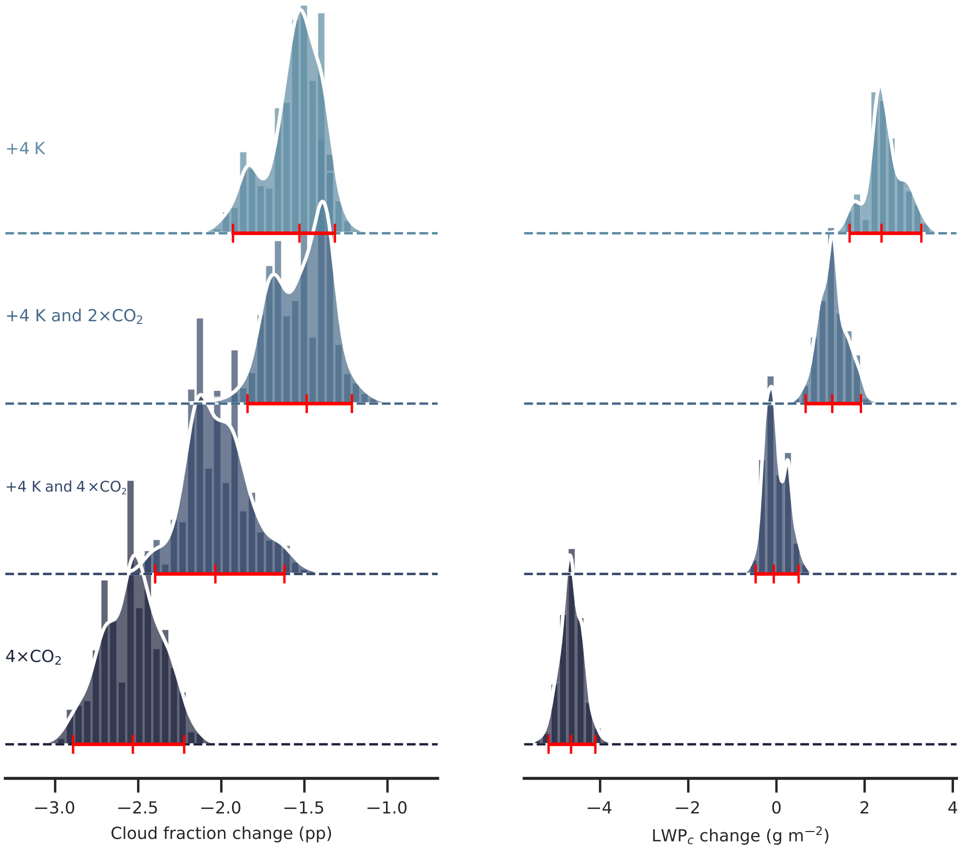

Fig. 4. Bootstrap distributions of median changes in marine low-cloud properties. Results are shown for the +4 K, +4 K + 2×CO_2, +4 K + 4×CO_2, and 4×CO_2 climate perturbation scenarios. All changes are calculated location-by-location and month-by-month relative to the AMIP baseline simulation. The 95% confidence interval for each distribution is indicated by a solid horizontal red line with end caps on its baseline, while the median is indicated by a central vertical tick on this line. (**Left panels**) Changes in cloud fraction (Δf_c, in percentage points). (**Right panels**) Changes in in-cloud liquid water path (ΔLWP_c, in g m^{-2}).

This more stable environment makes the cloud field more resilient to the direct cloud-dissipating effects of high CO_2 concentrations.

DISCUSSION

The strength of the low-cloud feedback is a primary determinant of ECS (*6*). Our LES-derived Pacific low-cloud feedback of 0.14 W m^{-2} K^{-1} under 4 K warming is consistent with recent observationally constrained global estimates (*9*). However, the total cloud radiative response strengthens to 0.43 W m^{-2} K^{-1} in the +4 K + 4×CO_2 scenario. This value is toward or beyond the upper end of most GCM simulations (*8*), suggesting that ECS may be considerably higher in high-CO_2 scenarios than many models project. This pronounced, state-dependent rapid cloud adjustment of low clouds to CO_2 may be key to understanding past hothouse climates such as the Eocene (*33*, *34*) and highlights the need to address known model biases in representing these clouds (*15*).

While offering helpful process insights, our findings are based on an idealized experimental design. Key limitations include large-scale conditions derived from a single GCM; an imposed uniform 4 K SST warming that bypasses the "pattern effect," in which feedback strength is sensitive to the spatial structure of surface warming (*6*, *10*, *40*–*42*); and fixed cloud droplet number concentrations, which excludes aerosol-cloud feedbacks such as interactions between higher in-cloud LWP and cloud droplet number concentration from enhanced precipitation. Nonetheless, these idealizations allow for a focused investigation of the specific temperature- and CO_2-driven mechanisms at the core of this study.

The extensive dataset from our LES, capturing thousands of low-cloud states, is publicly available. Because it systematically samples diverse meteorological conditions and varying baseline cloud states rather than relying on a few idealized cases, it offers a robust statistical sample of cloud states. This makes it a useful and potentially transformative resource for developing, training, and evaluating data-driven or next-generation turbulence, convection, and cloud schemes in global models. Last, while our study focuses strictly on marine low-level clouds, rapid cloud adjustments are known to be impactful over landmasses (*43*). Future large-ensemble LES work should investigate whether similar state-dependent nonlinearities exist in cloud regimes over land.

MATERIALS AND METHODS

LES model

Our LES code solves the anelastic equations for moist air (*26*), with prognostic equations for liquid-ice potential temperature (θ_{li}), total water specific humidity (q_t), and precipitation specific humidity (q_p). Given the focus of the simulations here on warm-cloud processes, q_p here represents rain specific humidity.

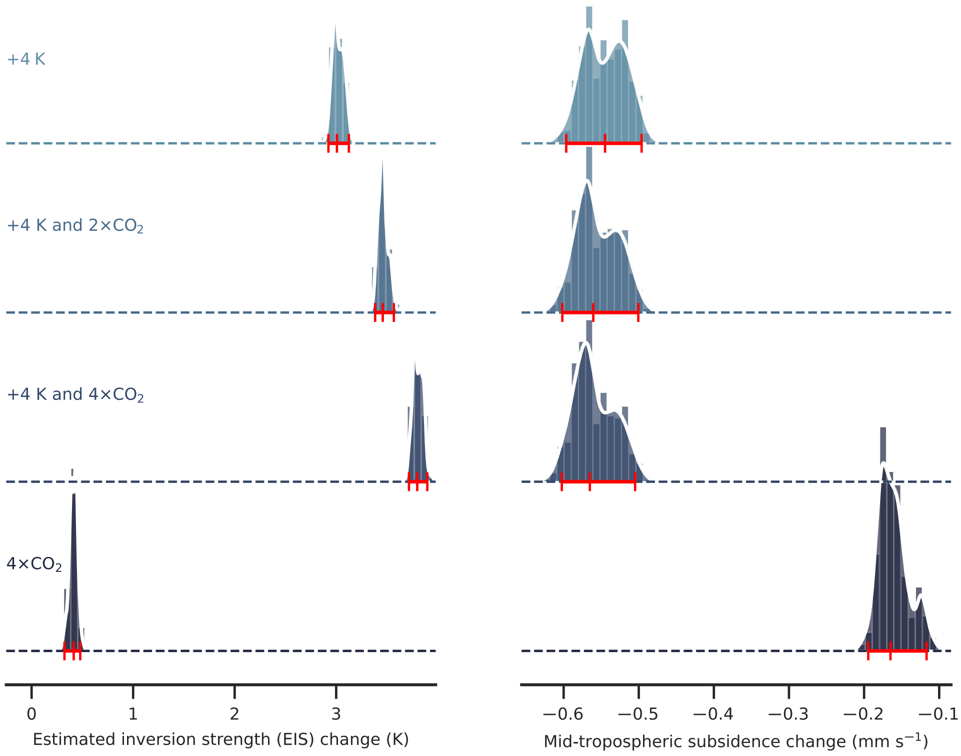

Fig. 5. Bootstrap distributions of median changes in key atmospheric environmental conditions. Results are shown for the +4 K, +4 K + 2×CO_2, +4 K + 4×CO_2, and 4×CO_2 climate perturbation scenarios. All changes are calculated relative to the AMIP baseline simulation for each corresponding location and month. The 95% confidence interval for each distribution is indicated by a solid horizontal red line with end caps on its baseline, while the median is indicated by a central vertical tick on this line. (**Left panels**) Change in EIS (*54*). (The EIS changes are similar to changes in lower-tropospheric stability.) (**Right panels**) Change in mid-tropospheric large-scale subsidence at 4-km altitude, with negative values indicating subsidence weakening.

Domain size and resolution

Each LES employs a vertical domain 6 km in height, capped by a no-slip, zero-flux lid at the top. The horizontal domain covers an area of $(6\ \text{km})^2$ and uses periodic lateral boundary conditions. Simulations are run for five simulated days on a computational grid of 128 by 128 by 480 points, with uniform horizontal and vertical grid spacings of 48.8 and 12.5 m, respectively. A physical time step of 0.2 s, corresponding to a Courant number of approximately 0.2, is used across all simulations. This domain size is chosen to resolve the dominant turbulent eddies governing low clouds, although it does not capture larger-scale mesoscale organization.

Microphysics

Microphysical processes such as autoconversion, accretion, and evaporation are parameterized largely following the single-moment warm rain bulk scheme based on the Kessler scheme (*44*) and detailed in (*45*, *46*). We adopt coefficients derived using an ensemble Kalman inversion (EKI) method to calibrate the scheme against an ensemble of superdroplet simulations (*46*). Our scheme assumes spherical cloud droplets with a fixed number concentration of $N_d = 10^8\ \text{m}^{-3}$.

There are two noteworthy departures from the scheme in (*45*, *46*). First, moist air is assumed to be in thermodynamic equilibrium, resulting in instantaneous condensation of supersaturated water vapor, rather than gradual condensation. This simplification is considered innocuous for our simulations with primarily warm clouds. Second, terminal velocity is parameterized separately: Instead of a power-law form for bulk fall speed, it is expressed as a linear combination of gamma-type functions (*47*). Last, cloud droplets within a given grid cell are assumed to be monodispersed, with their radius determined by the local humidity content and the fixed droplet concentration. Consequently, the bulk terminal velocity of cloud condensates simplifies to that of a monodispersed size distribution. While most of our simulations focus on warm cloud processes, some cases exhibit ice formation. The transition to the ice phase is modeled not as an abrupt cutoff at the freezing point, but with the liquid fraction represented as a continuous linear ramp between the homogeneous ice nucleation temperature (233 K) and the freezing point.

Radiative transfer

Radiative energy fluxes are calculated using the Rapid Radiative Transfer Model for GCMs (RRTMGP) (*48*), with fluxes updated every four simulated minutes. Atmospheric trace gas concentrations, excluding CO_2 and water vapor, are identical to those of the driving GCM. For gases with nonuniform vertical distributions, their GCM profiles are interpolated to the LES pressure grid. The H_2O volume mixing ratio is determined dynamically from the LES state, while CO_2 is assumed well-mixed with a uniform concentration, fixed to 400 parts per million in the baseline LES. Although the LES dynamic domain is 6 km deep, for radiative calculations with RRTMGP, the atmospheric column is effectively extended to the top of the driving GCM (approximately the 1-hPa level). This is achieved by interpolating the GCM's temperature and specific humidity profiles from all

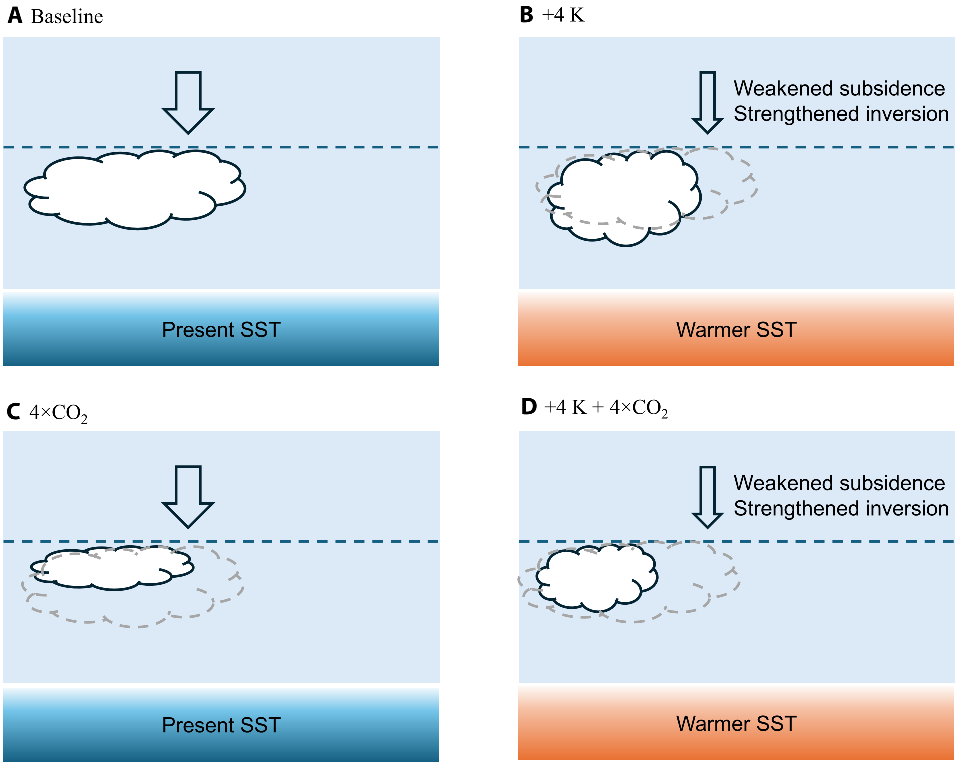

Fig. 6. Schematic representation of cloud responses to warming and increased CO_2. (**A**) Baseline climate with relatively strong large-scale subsidence (thick arrows) and a relatively weak inversion trapping marine boundary-layer clouds. (**B**) Higher SST (+4 K) leads to weaker large-scale subsidence (thin arrows) and a strengthened inversion, resulting in optically thicker clouds (higher in-cloud LWP) covering a smaller area. (**C**) Quadrupled CO_2 alone ($4\times CO_2$) has large-scale subsidence and inversion strength similar to the baseline, but the rapid adjustment to CO_2 markedly reduces in-cloud LWP and cloud fraction. (**D**) Combined forcing (+4 K + $4\times CO_2$) retains the weaker large-scale subsidence and strengthened inversion of the warmed climate, but the rapid CO_2 adjustments counteract the cloud thickening. This results in clouds covering a smaller area with an in-cloud LWP similar to the baseline, driving a strongly amplified positive feedback.

available GCM levels onto an extended grid above the LES domain. This extended portion, roughly seven times coarser than the LES vertical resolution, is kept cloud-free by design, meaning that radiative flux calculations exclude effects of any overlying GCM-generated high-level clouds from above the LES domain.

The radiative properties of simulated clouds are strongly influenced by the underlying microphysics scheme, which determines cloud particle characteristics (e.g., shape and size) and thereby cloud albedo. Given the assumptions of spherical cloud condensates and a fixed cloud droplet concentration, the cloud droplet effective radius for interpolating absorption coefficients is derived using a one-third power-law relationship with liquid water content (*49*), with an asymmetry factor of 0.8.

GCM forcing of LES

Following (*27*), our simulation setup aims to reproduce the thermodynamic and water budgets of a GCM grid column within an LES while minimally interfering with the LES dynamics. To this end, we prescribe 6-year averages of surface temperature, as well as GCM-derived large-scale horizontal advection and vertical transport (subsidence) as forcing in the LES. However, atmospheric temperature and moisture evolve freely within the high-resolution LES mesh, capturing important small-scale dynamical processes such as turbulence, convection, and clouds.

The forcing terms introduced into the LES prognostic equations for total water specific humidity and liquid-ice potential temperature are derived as follows. The GCM's specific humidity budget is given by

$$\frac{\partial \tilde{q}_t}{\partial t} + \tilde{u}\frac{\partial \tilde{q}_t}{\partial x} + \tilde{v}\frac{\partial \tilde{q}_t}{\partial y} + \frac{d\sigma}{dt}\frac{\partial \tilde{q}_t}{\partial \sigma} = J_q \tag{1}$$

where Cartesian coordinates are used for horizontal dimensions for simplicity. Tildes ($\tilde{\cdot}$) denote GCM variables and $\sigma = \tilde{p}/\tilde{p}_s$ is the GCM vertical coordinate (with pressure $\tilde{p}$ and surface pressure $\tilde{p}_s$). The right-hand side J_q represents the sum of all parameterized source terms for processes not resolved on the GCM grid (e.g., large-scale condensation).

In a statistically steady state, time-averaging this relation eliminates the explicit time derivative. The long-time mean advective tendency of total specific humidity is then

$$\langle \tilde{S}_{q,adv} \rangle = -\left\langle \tilde{u}\frac{\partial \tilde{q}_t}{\partial x} + \tilde{v}\frac{\partial \tilde{q}_t}{\partial y} \right\rangle - \left\langle \frac{d\sigma}{dt}\frac{\partial \tilde{q}_t}{\partial \sigma} \right\rangle \tag{2}$$

While the horizontal components of the advective tendency can be used directly in LES forcing, the GCM-resolved vertical advection (last term) requires modification to account for LES-resolved vertical eddies. This term is rewritten using the hydrostatic relation [approximating $(d\sigma/dt) \cdot \partial/\partial\sigma \approx w \cdot \partial/\partial z$] and decomposed into time-mean and fluctuating components

$$\left\langle \frac{\mathrm{d}\sigma}{\mathrm{d}t}\frac{\partial \tilde{q}_\mathrm{t}}{\partial \sigma}\right\rangle \approx \left\langle \tilde{w}\frac{\partial \tilde{q}_\mathrm{t}}{\partial z}\right\rangle = \left\langle \left(\langle \tilde{w}\rangle + \tilde{w}'\right)\partial_z\left(\left\langle \tilde{q}_\mathrm{t}\right\rangle + \tilde{q}'_\mathrm{t}\right)\right\rangle \approx \langle \tilde{w}\rangle \partial_z\left\langle \tilde{q}_\mathrm{t}\right\rangle + \left\langle \tilde{w}'\partial_z \tilde{q}'_\mathrm{t}\right\rangle \quad (3)$$

Here, $w = \mathrm{d}z/\mathrm{d}t$ is vertical velocity, $\langle\cdot\rangle$ denotes the temporal mean, and primes denote fluctuations about it. We estimate the time-averaged GCM vertical velocity $\langle \tilde{w}\rangle$ following (*50*), which derives it from the GCM pressure velocity via the hydrostatic relation and smoothly modulates its profile to ensure it vanishes at the surface.

Since the LES explicitly simulates its own vertical advection, we replace the mean GCM-resolved $\langle \tilde{q}_\mathrm{t}\rangle$ in the first term on the right of Eq. 3 (representing mean vertical advection by mean GCM winds) with the LES-resolved q_t. In the LES, the GCM forcing term for total specific humidity, S_q, thus becomes

$$S_\mathrm{q} = \left\langle \tilde{S}_\mathrm{q,adv}\right\rangle + \langle \tilde{w}\rangle\left(\partial_z\left\langle \tilde{q}_\mathrm{t}\right\rangle - \partial_z q_\mathrm{t}\right) \quad (4)$$

Note that while $\langle \tilde{S}_\mathrm{q,adv}\rangle$ is readily available from CMIP6 archives via time averaging, the time-averaged GCM vertical velocity $\langle \tilde{w}\rangle$ must be estimated as described above.

Similarly, the GCM temperature budget is

$$\frac{\partial \tilde{T}}{\partial t} + \tilde{u}\frac{\partial \tilde{T}}{\partial x} + \tilde{v}\frac{\partial \tilde{T}}{\partial y} + \frac{\mathrm{d}\sigma}{\mathrm{d}t}\frac{\partial \tilde{T}}{\partial \sigma} - \frac{\tilde{\alpha}\tilde{\omega}}{c_\mathrm{p}} = J_\mathrm{T} \quad (5)$$

where $\tilde{\alpha}$ is GCM specific volume, c_p is the isobaric specific heat capacity of dry air, $\tilde{\omega} = \mathrm{d}\tilde{p}/\mathrm{d}t$, and J_T includes all parameterized heat sources. The pressure-volume work term can be approximated using the hydrostatic approximation as

$$-\frac{\tilde{\alpha}\tilde{\omega}}{c_\mathrm{p}} \approx \frac{g\langle \tilde{w}\rangle}{c_\mathrm{p}} \quad (6)$$

where g is gravitational acceleration. The temperature source term for the LES, S_T, is derived analogously to S_q, using the LES temperature T

$$S_\mathrm{T} = \left\langle \tilde{S}_\mathrm{T,adv}\right\rangle + \langle \tilde{w}\rangle\left(\partial_z\left\langle \tilde{T}\right\rangle - \partial_z T\right) \quad (7)$$

Here, $\langle \tilde{S}_\mathrm{T,adv}\rangle$ is the GCM-resolved advective tendency of temperature, which is also readily available from CMIP6 archives.

Because the LES prognostic energy equation uses liquid-ice potential temperature (θ_li), S_T is transformed to the source term S_θ via

$$S_\theta \approx \frac{1}{\Pi}\left(\left\langle \tilde{S}_\mathrm{T,adv}\right\rangle + \langle \tilde{w}\rangle\partial_z\left\langle \tilde{T}\right\rangle + \frac{g\langle \tilde{w}\rangle}{c_\mathrm{p}}\right) - \langle \tilde{w}\rangle\partial_z\theta_\mathrm{li} \quad (8)$$

where Π is the Exner function. Since the advection term involving the LES liquid-ice potential temperature θ_li implicitly includes the LES pressure-volume work, the GCM mean pressure-volume work (Eq. 6) is subtracted in the source.

Nudging and sponge layer

To prevent model drift from the large-scale state imposed by the GCM, a relaxation scheme is adopted. Horizontal momentum is nudged toward mean GCM profiles across the entire domain with a 6-hour timescale. In the free troposphere (above $z_i = 4$ km), q_t and θ_li are also relaxed to GCM profiles using a height-dependent timescale similar to the one in (*27*), but with a relaxation constant (τ_r) of 6 hours and a more gradual ramp-up over a 2-km vertical height range. In addition, a sponge layer in the upper 1 km of the domain, implemented as a linear Rayleigh damping layer (*51*), relaxes vertical velocity to zero to absorb upward-propagating waves.

Initial conditions and reference state

Initial conditions for the LES are 6-year GCM column state averages for the given month, preprocessed to convert mean temperature to potential temperature and to interpolate GCM vertical levels to the 12.5-m vertical LES resolution. The LES reference pressure profile (p_0) specific for each location, month, and scenario is constructed from the GCM mean virtual temperature profile $\langle T_\mathrm{v}\rangle$ using the hypsometric equation, enforcing hydrostatic balance discretely by using a trapezoidal rule for the integration

$$p_0(z) = p_\mathrm{s}\exp\left(-\frac{g}{R_\mathrm{d}}\int_0^z \frac{\mathrm{d}z'}{\langle T_\mathrm{v}(z')\rangle}\right) \quad (9)$$

where p_s is the GCM surface pressure. This p_0 profile is used in all thermodynamic calculations.

Lower boundary condition and radiative parameters

Each LES emulates the local GCM climate: SST, diurnally averaged TOA insolation, and insolation-weighted zenith angle are set to their GCM-derived monthly means for the given location and month. Applying a constant diurnally-averaged forcing rather than an explicit diurnal cycle allows the LES to reach a stable quasi-steady state by the fifth day, preventing transient diurnal effects from complicating the extraction of the mean thermodynamic cloud feedback signal. Radiative transfer is then solved using the two-stream RRTMGP algorithm (*48*). This code produces upwelling and downwelling radiative fluxes at all grid cell interfaces. The net flux divergence provides the local radiative heating rates. Fluxes are updated every four simulated minutes, balancing computational cost with the relatively slow changes in mean radiative profiles.

Experimental design

Our experimental framework expands on (*27*) using thousands of high-resolution simulations driven by large-scale GCM forcings to investigate low-cloud regions (cloud top height < 4 km) over the Pacific. We use 6-year (2009–2014) averaged large-scale forcings derived from the NOAA-GFDL CM4 model output for four representative months (January, April, July, and October). Figure S1 shows representative mid-tropospheric vertical velocities in the GCM and in reanalysis data, demonstrating that they compare well, with some notable differences in the extent of the strong subsidence region in the northeastern subtropical Pacific; such large-scale differences may in part be responsible for the differences between LES-simulated and observed cloud cover statistics (figs. S2 to S7).

Locations were selected from the tropical Pacific (35°S to 35°N) by first filtering based on ERA5 reanalysis data (*52*) to eliminate sites with weak subsidence or ascent (time-averaged mid-tropospheric pressure velocities below 8 hPa day^{-1}). This filter removed sites where conditions were unfavorable for sustaining low clouds or were favorable for deep convection. From the remaining candidate locations, 500 distinct sites were selected by sampling from a unique low-cloud cover distribution obtained from annually averaged CloudSat and CALIPSO satellite data (*31*) interpolated to the GCM grid. This process yielded a subset of low-cloud regions representative of the observed distribution of tropical marine low-cloud regimes.

We initially generated large-scale forcings for 500 candidate locations across 4 months for five scenarios, totaling 10,000 simulations. From this total pool, we excluded simulations on an individual basis if clouds grew above a 4-km altitude. To ensure robust comparative statistics and avoid sampling bias, our analyses of changes (e.g., Δf_c, ΔLWP_c, and cloud feedback) were strictly performed pairwise: We only included a location and month in a specific perturbation scenario if both the baseline run and the perturbed run successfully stayed below the 4-km threshold. This filtering yielded the final set of 7083 LESs used for the analysis. However, we acknowledge that it may introduce a subtle selection bias by filtering situations more prone to deep convection.

The five scenarios used the following forcings: The baseline LES was driven by time-averaged large-scale forcings from the standard AMIP configuration, representing the present-day climate. Forcing for the primary warming scenario was derived from the AMIP-P4K configuration, which uniformly increases historical SST by 4 K. Forcing for a scenario investigating rapid cloud adjustment to CO_2 uses the AMIP-4×CO_2 configuration, which quadruples CO_2 concentration relative to the baseline. Two additional scenarios combined the large-scale forcings of the AMIP-P4K model with either doubled or quadrupled atmospheric CO_2 concentrations.

CRE and feedback calculation

The CRE is defined as the difference between the TOA radiative fluxes for the full sky and the TOA radiative fluxes for an otherwise identical but cloud-free scene. Changes in CRE (ΔCRE) between experiments are calculated as the difference between the CRE of a perturbed simulation and that of its corresponding baseline simulation.

To accurately isolate the cloud feedback, we use the PRP method, which avoids the systematic biases, for example, due to differential effects of water vapor concentration changes in cloudy and clear regions ("cloud masking") that are inherent in direct calculations of CRE changes (*53*). The PRP method determines the radiative impact arising exclusively from changes in cloud properties between a baseline (AMIP-driven) and a perturbed (e.g., AMIP-P4K–driven) simulation. This requires offline radiative transfer calculations on the archived simulation output. Specifically, for a given baseline atmospheric state (temperature field T, specific humidity field q_v), the cloud water mass fraction field (q_c) from the final snapshot of the baseline LES is replaced by the corresponding field from a perturbed simulation (q_c'). The net TOA absorbed radiation (R) is computed for both the original and cloud-perturbed states using the same radiative transfer code as the online simulation. The horizontally averaged anomaly in this net radiation, normalized by the prescribed SST perturbation (ΔT_s), yields the forward cloud feedback ($\lambda_{c,f}$)

$$\lambda_{c,f} = \frac{\overline{R}(T, q_v, q_c') - \overline{R}(T, q_v, q_c)}{\Delta T_s} \tag{10}$$

Here, $R = \text{ASR} - \text{OLR}$ (where ASR is the absorbed shortwave radiation and OLR is the outgoing longwave radiation), and the overbar indicates a horizontal average at the TOA. For our analysis, this calculation is performed for each component of R to decompose the feedback into its SW and LW parts. To avoid state-dependent biases inherent in evaluating finite perturbations, we also performed a backward PRP calculation, substituting the baseline cloud fields into the perturbed atmospheric state to obtain the backward cloud feedback $\lambda_{c,b}$

$$\lambda_{c,b} = \frac{\overline{R}(T', q_v', q_c') - \overline{R}(T', q_v', q_c)}{\Delta T_s} \tag{11}$$

where primes denote the corresponding fields from the perturbed simulation. The final net, SW, and LW cloud feedback values reported in our analysis are the average of the forward and backward PRP calculations.

For these offline calculations, thermodynamic consistency is maintained by adjusting the total specific humidity (q_t) to be the sum of the original water vapor specific humidity (q_v) and the newly substituted cloud water specific humidity (q_c'). While q_v is held fixed, the change in q_t alters the water vapor volume mixing ratio (VMR_{H_2O}), a key input for the RRTMGP radiation code, computed as

$$VMR_{H_2O} = \frac{R_d}{R_v} \frac{q_v}{1 - q_t} \tag{12}$$

where R_d and R_v are the specific gas constants for dry air and water vapor. This coupling between the cloud water change and VMR_{H_2O} means that a reduction in cloud water slightly decreases the water vapor VMR (a greenhouse gas), which could introduce small changes in the calculated SW and LW feedbacks. However, sensitivity tests where we held VMR_{H_2O} fixed confirmed this effect is negligible, producing statistically equivalent feedback results.

This feedback calculation is applied to each of the three AMIP-P4K experiment variants (with present-day, doubled, and quadrupled CO_2 concentrations), yielding three distinct total cloud radiative response values for each location and month.

Uncertainty estimation

In addition to cloud feedbacks, we analyzed changes across experiments for key cloud and environmental properties, including CRE, liquid water path (LWP), cloud fraction (f_c), cloud top height, EIS, and subsidence. Cloud fraction was computed as the fraction of total grid columns in our LES that are not entirely cloud-free. A column is flagged as "cloudy" if any grid cell within it (at any height) contains a nonzero cloud condensate mass fraction. For each simulation, these properties were averaged over the fifth and final simulated day, by which point the LES had reached a quasi-steady state.

To quantify the uncertainty of our ensemble-wide results, we created bootstrap distributions for the median of all derived quantities (i.e., net, SW, and LW total cloud radiative responses, and changes in cloud and environmental properties). The median was chosen for its robustness against outliers. Each bootstrap distribution was generated by resampling with replacement from the full ensemble of simulation results 10,000 times. From the resulting distribution of these 10,000 sample medians, we then computed the 95% confidence interval as the interval from the 2.5th to the 97.5th percentile to represent the uncertainty for each variable, as presented in our results.

Supplementary Materials

This PDF file includes:
Figs. S1 to S15
References

REFERENCES

1. F. A.-M. Bender, R. J. Charlson, A. M. Ekman, L. V. Leahy, Quantification of monthly mean regional-scale albedo of marine stratiform clouds in satellite observations and GCMs. *J. Appl. Meteorol. Climatol.* **50**, 2139–2148 (2011).

2. S. Bony, J. L. Dufresne, Marine boundary layer clouds at the heart of tropical cloud feedback uncertainties in climate models. *Geophys. Res. Lett.* **32**, L20806 (2005).
3. J. Vial, J.-L. Dufresne, S. Bony, On the interpretation of inter-model spread in CMIP5 climate sensitivity estimates. *Climate Dynam.* **41**, 3339–3362 (2013).
4. T. Schneider, J. Teixeira, C. S. Bretherton, F. Brient, K. G. Pressel, C. Schär, A. P. Siebesma, Climate goals and computing the future of clouds. *Nat. Clim. Chang.* **7**, 3–5 (2017).
5. G. L. Stephens, Cloud feedbacks in the climate system: A critical review. *J. Climate* **18**, 237–273 (2005).
6. S. C. Sherwood, M. J. Webb, J. D. Annan, K. C. Armour, P. M. Forster, J. C. Hargreaves, G. Hegerl, S. A. Klein, K. D. Marvel, E. J. Rohling, M. Watanabe, T. Andrews, P. Braconnot, C. S. Bretherton, G. L. Foster, Z. Hausfather, A. S. von der Heydt, R. Knutti, T. Mauritsen, J. R. Norris, C. Proistosescu, M. Rugenstein, G. A. Schmidt, K. B. Tokarska, M. D. Zelinka, An assessment of Earth's climate sensitivity using multiple lines of evidence. *Rev. Geophys.* **58**, e2019RG000678 (2020).
7. P. Forster, T. Storelvmo, K. Armour, W. Collins, J.-L. Dufresne, D. Frame, D.J. Lunt, T. Mauritsen, M.D. Palmer, M. Watanabe, M. Wild, H. Zhang, The Earth's energy budget, climate feedbacks, and climate sensitivity, in *Climate Change 2021: The Physical Science Basis. Contribution of Working Group I to the Sixth Assessment Report of the Intergovernmental Panel on Climate Change*, V. Masson-Delmotte, P. Zhai, A. Pirani, S.L. Connors, C. Péan, S. Berger, N. Caud, Y. Chen, L. Goldfarb, M.I. Gomis, M. Huang, K. Leitzell, E. Lonnoy, J.B.R. Matthews, T.K. Maycock, T. Waterfield, O. Yelekçi, R. Yu, B. Zhou, Eds. (Cambridge Univ. Press, 2021), pp. 923–1054.
8. M. D. Zelinka, T. A. Myers, D. T. McCoy, S. Po-Chedley, P. M. Caldwell, P. Ceppi, S. A. Klein, K. E. Taylor, Causes of higher climate sensitivity in CMIP6 models. *Geophys. Res. Lett.* **47**, e2019GL085782 (2020).
9. T. A. Myers, R. C. Scott, M. D. Zelinka, S. A. Klein, J. R. Norris, P. M. Caldwell, Observational constraints on low cloud feedback reduce uncertainty of climate sensitivity. *Nat. Clim. Chang.* **11**, 501–507 (2021).
10. G. V. Cesana, A. D. Del Genio, Observational constraint on cloud feedbacks suggests moderate climate sensitivity. *Nat. Clim. Chang.* **11**, 213–218 (2021).
11. M. D. Zelinka, S. A. Klein, Y. Qin, T. A. Myers, Evaluating climate models' cloud feedbacks against expert judgment. *J. Geophys. Res. Atmos.* **127**, e2021JD035198 (2022).
12. F. Brient, T. Schneider, Z. Tan, S. Bony, X. Qu, A. Hall, Shallowness of tropical low clouds as a predictor of climate models' response to warming. *Climate Dynam.* **47**, 433–449 (2016).
13. P. M. Caldwell, M. D. Zelinka, K. E. Taylor, K. Marvel, Quantifying the sources of intermodel spread in equilibrium climate sensitivity. *J. Climate* **29**, 513–524 (2016).
14. L.-W. Chao, M. D. Zelinka, A. E. Dessler, Evaluating cloud feedback components in observations and their representation in climate models. *J. Geophys. Res. Atmos.* **129**, e2023JD039427 (2024).
15. P. Ceppi, T. A. Myers, P. Nowack, C. J. Wall, M. D. Zelinka, Implications of a pervasive climate model bias for low-cloud feedback. *Geophys. Res. Lett.* **51**, e2024GL110525 (2024).
16. M. Zhang, C. S. Bretherton, P. N. Blossey, P. H. Austin, J. T. Bacmeister, S. Bony, F. Brient, S. K. Cheedela, A. Cheng, A. D. Del Genio, S. R. de Roode, S. Endo, C. N. Franklin, J. C. Golaz, C. Hannay, T. Heus, F. A. Isotta, J. L. Dufresne, I. S. Kang, H. Kawai, M. Köhler, V. E. Larson, Y. Liu, A. P. Lock, U. Lohmann, M. F. Khairoutdinov, A. M. Molod, R. A. J. Neggers, P. Rasch, I. Sandu, R. Senkbeil, A. P. Siebesma, C. Siegenthaler-le Drian, B. Stevens, M. J. Suarez, K. M. Xu, K. von Salzen, M. J. Webb, A. Wolf, M. Zhao, CGILS: Results from the first phase of an international project to understand the physical mechanisms of low cloud feedbacks in general circulation models. *J. Adv. Model. Earth Syst.* **5**, 826–842 (2013).
17. C. S. Bretherton, P. N. Blossey, C. R. Jones, Mechanisms of marine low cloud sensitivity to idealized climate perturbations: A single-LES exploration extending the CGILS cases. *J. Adv. Model. Earth Syst.* **5**, 316–337 (2013).
18. P. N. Blossey, C. S. Bretherton, A. Cheng, S. Endo, T. Heus, A. P. Lock, J. J. van der Dussen, CGILS Phase 2 LES intercomparison of response of subtropical marine low cloud regimes to CO_2 quadrupling and a CMIP3 composite forcing change. *J. Adv. Model. Earth Syst.* **8**, 1714–1726 (2016).
19. Z. Tan, T. Schneider, J. Teixeira, K. G. Pressel, Large-eddy simulation of subtropical cloud-topped boundary layers: 2. Cloud response to climate change. *J. Adv. Model. Earth Syst.* **9**, 19–38 (2017).
20. P. G. Hill, D. L. Finney, M. D. Zelinka, Cloud feedback uncertainty in the equatorial Pacific across CMIP6 models. *Geophys. Res. Lett.* **52**, e2025GL117183 (2025).
21. J. Gregory, M. Webb, Tropospheric adjustment induces a cloud component in CO_2 forcing. *J. Clim.* **21**, 58–71 (2008).
22. M. D. Zelinka, S. A. Klein, K. E. Taylor, T. Andrews, M. J. Webb, J. M. Gregory, P. M. Forster, Contributions of different cloud types to feedbacks and rapid adjustments in CMIP5. *J. Climate* **26**, 5007–5027 (2013).
23. C. S. Bretherton, P. N. Blossey, Low cloud reduction in a greenhouse-warmed climate: Results from Lagrangian LES of a subtropical marine cloudiness transition. *J. Adv. Model. Earth Syst.* **6**, 91–114 (2014).
24. C. S. Bretherton, Insights into low-latitude cloud feedbacks from high-resolution models. *Philos. Trans. A Math. Phys. Eng. Sci.* **373**, 20140415 (2015).
25. M. Wu, H. Su, J. D. Neelin, Multi-objective observational constraint of tropical Atlantic and Pacific low-cloud variability narrows uncertainty in cloud feedback. *Nat. Commun.* **16**, 218 (2025).
26. S. Chammas, Q. Wang, T. Schneider, M. Ihme, Y.-f. Chen, J. Anderson, Accelerating large-eddy simulations of clouds with tensor processing units. *J. Adv. Model. Earth Syst.* **15**, e2023MS003619 (2023).
27. Z. Shen, A. Sridhar, Z. Tan, A. Jaruga, T. Schneider, A library of large-eddy simulations forced by global climate models. *J. Adv. Model. Earth Syst.* **14**, e2021MS002631 (2022).
28. M. Zhao, J.-C. Golaz, I. M. Held, H. Guo, V. Balaji, R. Benson, J.-H. Chen, X. Chen, L. J. Donner, J. P. Dunne, K. Dunne, J. Durachta, S.-M. Fan, S. M. Freidenreich, S. T. Garner, P. Ginoux, L. M. Harris, L. W. Horowitz, J. P. Krasting, A. R. Langenhorst, Z. Liang, P. Lin, S.-J. Lin, S. L. Malyshev, E. Mason, P. C. D. Milly, Y. Ming, V. Naik, F. Paulot, D. Paynter, P. Phillipps, A. Radhakrishnan, V. Ramaswamy, T. Robinson, D. Schwarzkopf, C. J. Seman, E. Shevliakova, Z. Shen, H. Shin, L. G. Silvers, J. R. Wilson, M. Winton, A. T. Wittenberg, B. Wyman, B. Xiang, The GFDL global atmosphere and land model AM4.0/LM4.0: 1. Simulation characteristics with prescribed SSTs. *J. Adv. Model. Earth Syst.* **10**, 691–734 (2018).
29. M. Zhao, J.-C. Golaz, I. M. Held, H. Guo, V. Balaji, R. Benson, J.-H. Chen, X. Chen, L. J. Donner, J. P. Dunne, K. Dunne, J. Durachta, S.-M. Fan, S. M. Freidenreich, S. T. Garner, P. Ginoux, L. M. Harris, L. W. Horowitz, J. P. Krasting, A. R. Langenhorst, Z. Liang, P. Lin, S.-J. Lin, S. L. Malyshev, E. Mason, P. C. D. Milly, Y. Ming, V. Naik, F. Paulot, D. Paynter, P. Phillipps, A. Radhakrishnan, V. Ramaswamy, T. Robinson, D. Schwarzkopf, C. J. Seman, E. Shevliakova, Z. Shen, H. Shin, L. G. Silvers, J. R. Wilson, M. Winton, A. T. Wittenberg, B. Wyman, B. Xiang, The GFDL global atmosphere and land model AM4.0/LM4.0: 2. Model description, sensitivity studies, and tuning strategies. *J. Adv. Model. Earth Syst.* **10**, 735–769 (2018).
30. K. A. Schiro, H. Su, F. Ahmed, N. Dai, C. E. Singer, P. Gentine, G. S. Elsaesser, J. H. Jiang, Y.-S. Choi, J. David Neelin, Model spread in tropical low cloud feedback tied to overturning circulation response to warming. *Nat. Commun.* **13**, 7119 (2022).
31. W. Bertrand, J. E. Kay, J. Haynes, G. de Boer, A global gridded dataset for cloud vertical structure from combined CloudSat and CALIPSO observations. *Earth Syst. Sci. Data* **16**, 1301–1316 (2024).
32. R. C. Scott, T. A. Myers, J. R. Norris, M. D. Zelinka, S. A. Klein, M. Sun, D. R. Doelling, Observed sensitivity of low-cloud radiative effects to meteorological perturbations over the global oceans. *J. Climate* **33**, 7717–7734 (2020).
33. G. L. Foster, D. L. Royer, D. J. Lunt, Future climate forcing potentially without precedent in the last 420 million years. *Nat. Commun.* **8**, 14845 (2017).
34. E. J. Judd, J. E. Tierney, D. J. Lunt, I. P. Montañez, B. T. Huber, S. L. Wing, P. J. Valdes, A 485-million-year history of Earth's surface temperature. *Science* **385**, eadk3705 (2024).
35. R. T. Wetherald, S. Manabe, Cloud feedback processes in a general circulation model. *J. Atmos. Sci.* **45**, 1397–1416 (1988).
36. B. J. Soden, I. M. Held, R. Colman, K. M. Shell, J. T. Kiehl, C. A. Shields, Quantifying climate feedbacks using radiative kernels. *J. Clim.* **21**, 3504–3520 (2008).
37. L. Peng, P. N. Blossey, W. M. Hannah, C. S. Bretherton, C. R. Terai, A. M. Jenney, S. L. Ferretti, H. Parishani, M. S. Pritchard, Resolving low cloud feedbacks globally with E3SM High-Res MMF: Agreement with LES but stronger shortwave effects. *J. Adv. Model. Earth Syst.* **17**, e2025MS005003 (2025).
38. T. Schneider, C. M. Kaul, K. G. Pressel, Possible climate transitions from breakup of stratocumulus decks under greenhouse warming. *Nat. Geosci.* **12**, 163–167 (2019).
39. T. Schneider, C. M. Kaul, K. G. Pressel, Solar geoengineering may not prevent strong warming from direct effects of CO_2 on stratocumulus cloud cover. *Proc. Natl. Acad. Sci. U.S.A.* **117**, 30179–30185 (2020).
40. M. Winton, K. Takahashi, I. M. Held, Importance of ocean heat uptake efficacy to transient climate change. *J. Clim.* **23**, 2333–2344 (2010).
41. T. Andrews, J. M. Gregory, D. Paynter, L. G. Silvers, C. Zhou, T. Mauritsen, M. J. Webb, K. C. Armour, P. M. Forster, H. Titchner, Accounting for changing temperature patterns increases historical estimates of climate sensitivity. *Geophys. Res. Lett.* **45**, 8490–8499 (2018).
42. T. A. Myers, M. D. Zelinka, S. A. Klein, Observational constraints on the cloud feedback pattern effect. *J. Climate* **36**, 6533–6545 (2023).
43. Y. Kamae, M. Watanabe, T. Ogura, M. Yoshimori, H. Shiogama, Rapid adjustments of cloud and hydrological cycle to increasing CO_2: A review. *Curr. Clim. Change Rep.* **1**, 103–113 (2015).
44. E. Kessler, *On the Distribution and Continuity of Water Substance in Atmospheric Circulations* (American Meteorological Society, 1969), vol. 10.
45. Climate Modeling Alliance, CloudMicrophysics.jl [Software] (GitHub, 2025); https://github.com/CliMA/CloudMicrophysics.jl.
46. S. Azimi, A. Jaruga, E. K. de Jong, S. Arabas, T. Schneider, Training warm-rain bulk microphysics schemes using super-droplet simulations. *J. Adv. Model. Earth Syst.* **16**, e2023MS004028 (2024).
47. J.-P. Chen, T.-W. Hsieh, Y.-C. Lin, C.-K. Yu, Accurate parameterization of precipitation particles' fall speeds for bulk cloud microphysics schemes. *Atmos. Res.* **273**, 106171 (2022).
48. R. Pincus, E. J. Mlawer, J. S. Delamere, Balancing accuracy, efficiency, and flexibility in radiation calculations for dynamical models. *J. Adv. Model. Earth Syst.* **11**, 3074–3089 (2019).

49. Y. Liu, J. Hallett, The '1/3' power law between effective radius and liquid-water content. *Q. J. R. Meteorol. Soc.* **123**, 1789–1795 (1997).
50. R. L. Atlas, C. S. Bretherton, P. N. Blossey, A. Gettelman, C. Bardeen, P. Lin, Y. Ming, How well do large-eddy simulations and global climate models represent observed boundary layer structures and low clouds over the summertime Southern Ocean? *J. Adv. Model. Earth Syst.* **12**, e2020MS002205 (2020).
51. D. R. Durran, J. B. Klemp, A compressible model for the simulation of moist mountain waves. *Mon. Weather Rev.* **111**, 2341–2361 (1983).
52. H. Hersbach, B. Bell, P. Berrisford, S. Hirahara, A. Horányi, J. Muñoz-Sabater, J. Nicolas, C. Peubey, R. Radu, D. Schepers, A. Simmons, C. Soci, S. Abdalla, X. Abellan, G. Balsamo, P. Bechtold, G. Biavati, J. Bidlot, M. Bonavita, G. de Chiara, P. Dahlgren, D. Dee, M. Diamantakis, R. Dragani, J. Flemming, R. Forbes, M. Fuentes, A. Geer, L. Haimberger, S. Healy, R. J. Hogan, E. Hólm, M. Janisková, S. Keeley, P. Laloyaux, P. Lopez, C. Lupu, G. Radnoti, P. de Rosnay, I. Rozum, F. Vamborg, S. Villaume, J.-N. Thépaut, The ERA5 global reanalysis. *Q. J. R. Meteorol. Soc.* **146**, 1999–2049 (2020).
53. B. J. Soden, A. J. Broccoli, R. S. Hemler, On the use of cloud forcing to estimate cloud feedback. *J. Clim.* **17**, 3661–3665 (2004).
54. R. Wood, C. S. Bretherton, On the relationship between stratiform low cloud cover and lower-tropospheric stability. *J. Clim.* **19**, 6425–6432 (2006).
55. G. S. Elsaesser, C. W. O'Dell, M. D. Lebsock, R. Bennartz, T. J. Greenwald, F. J. Wentz, The Multisensor Advanced Climatology of Liquid Water Path (MAC-LWP). *J. Clim.* **30**, 10193–10210 (2017).
56. N. G. Loeb, D. R. Doelling, H. Wang, W. Su, C. Nguyen, J. G. Corbett, L. Liang, C. Mitrescu, F. G. Rose, S. Kato, Clouds and the Earth's Radiant Energy System (CERES) Energy Balanced and Filled (EBAF) top-of-atmosphere (TOA) Edition-4.0 data product. *J. Climate* **31**, 895–918 (2018).

Acknowledgments: We thank A. Boral, J. Lottes, R. Larsen, and S. Agarwal for the technical help in the LES development; T. Russell and C. Bromberg for the technical program management; and C. Cortes, Y. Matias, F. Pereira, and J. Platt for the leadership support. **Funding:** This work was supported by the National Science Foundation (NSF) Award AGS-1835860 (to Z.S.) and Schmidt Sciences LLC (to Z.S.). **Author contributions:** Conceptualization: T.S. Methodology: S.C. and T.S. Software: S.C., Q.W., T.S., J.B.P., C.G., M.I., and Y.-F.C. Validation: S.C., Q.W., T.S., R.C., Z.S., and J.B.P. Formal analysis: S.C., T.S., and Q.W. Investigation: S.C., T.S., R.C., J.B.P., and Y.-F.C. Resources: Y.-F.C., and J.A. Data curation: S.C. and Z.S. Writing—Original draft: T.S. and S.C. Writing—Review and editing: Q.W., R.C., Z.S., J.B.P., C.G., M.I., Y.-F.C., T.S., and S.C. Visualization: S.C., Q.W., C.G., and Z.S. Project administration: Y.-F.C. Supervision: Y.-F.C. and J.A. Funding acquisition: T.S. **Competing interests:** S.C., Q.W., T.S., J.B.P., C.G., M.I., Y.-F.C, R.C., and J.A. are employees of Google LLC and own Alphabet stock as part of the standard compensation package. All other authors declare that they have no competing interests. **Data, code, and materials availability:** We thank the climate modeling groups for producing and making available their model output, which is accessible on the Earth System Grid Federation (ESGF) Portal (https://esgf-node.llnl.gov/search/cmip6/). All code and data needed to replicate the results in this paper are publicly available. The code for replication of the simulations and analyses has been permanently archived on Zenodo (https://zenodo.org/records/19561733), with the active repository available as an additional resource at https://github.com/google-research/swirl-lm. The post-processed simulation statistics needed to replicate the analyses are publicly available at https://doi.org/10.22002/hgpq9-a1861. In addition, the raw LES output from which the statistics are derived (203.78 terabyte of data) is accessible via Google Cloud Storage at https://storage.googleapis.com/cloudbench-simulation-output/index.html. Further details of how to access it can be found at https://github.com/google-research/swirl-lm/tree/main/swirl_lm/example/geo_flows/cloud_feedback/README.md. This study did not generate new materials other than the code and data provided here.

Submitted 6 October 2025
Accepted 15 June 2026
Published 24 July 2026
10.1126/sciadv.aec8488
